# Factors influencing range contraction of a rodent herbivore in a steppe grassland over the past decades

**DOI:** 10.1002/ece3.8546

**Published:** 2022-02-14

**Authors:** Defeng Bai, Xinru Wan, Guoliang Li, Xinrong Wan, Yongwang Guo, Dazhao Shi, Zhibin Zhang

**Affiliations:** ^1^ State Key Laboratory of Integrated Management of Pest Insects and Rodents Institute of Zoology Chinese Academy of Sciences Beijing China; ^2^ CAS Center for Excellence in Biotic Interactions University of Chinese Academy of Sciences Beijing China; ^3^ National Agro‐tech Extension and Service Center Beijing China; ^4^ College of Plant Protection China Agricultural University Beijing China

**Keywords:** Brandt's vole, climate change, human disturbance, local disappearance, range contraction, steppe grassland

## Abstract

1. Climate warming and human disturbance are known to be key drivers in causing range contraction of many species, but quantitative assessment on their distinctive and interactive effects on local disappearance is still rare.
2. In this study, we examined the association of climate warming and human disturbance stressors with local disappearance probability of Brandt's voles (*Lasiopodomys brandtii*) in a steppe grassland in northern China.
3. We used logistic generalized additive models to quantify the relationship between local disappearance probability of Brandt's voles and environmental variables. The year following the last observation year was used to estimate the disappearance threshold of Brandt's voles. We projected the distribution change of Brandt's voles under future climate warming scenarios.
4. We found climate warming attributed to local disappearance and range contraction for southern populations of Brandt's voles from 1971 to 2020. Human stressors and high vegetation coverage increased the probability of local disappearance of voles in years of abundant precipitation. The southern boundary retreated northward at a speed of 99.0 km per decade with the temperature rise of 0.36°C. The disappearance threshold of maximum air temperature of Brandt's voles in the warmest month (27.50 ± 1.61°C) was similar to the lower critical temperature of its thermal neutral zone.
5. Our study suggests that the rapid climate change over the past decades contributed to the range contraction of its southern boundary of this keystone species in the steppe grassland of China. It is necessary to take actions to preserve the isolated populations of Brandt's voles from the effects of accelerated climate change and human disturbance.

## Graphical abstract

We found climate warming attributed to local disappearance and range contraction for southern populations of Brandt's voles from 1971 to 2020. The southern boundary of Brandt's voles retreated toward the north pole at a speed of 99.0 km per decade with the temperature rise of 0.36°C.

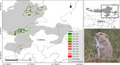

## 1 INTRODUCTION

Currently, the earth is facing rapid biodiversity loss, which is expected to have profound consequences on ecosystem services and functions (Sintayehu, 2018). Rates of population decline of various taxa have increased during the past half century and species diversity loss is expected to continue by 2100 in different ecosystems around the world (Butchart et al., 2010; Sala et al., 2000). For mammal species, approximately half of them are declining in population abundance and some species have shown a large‐scale range contraction (Schipper et al., 2008).

Climate change has been widely reported to cause range shifts and contraction of animal populations (Chen et al., 2011). An assessment of 526 populations of 190 mammal species indicates that rapid climate warming is associated with mammal population decline globally since 1950 (Spooner et al., 2018). Local extinction rates of mammals are anticipated to increase with global warming in the future (Moritz & Agudo, 2013). Among mammals, in the Northern Great Lakes Region, USA, four rodent species in low‐latitude areas expanded their distribution, but five rodent species in higher latitudes showed range contraction due to climate warming from 1883 to 2006 (Myers et al., 2009). In Yosemite National Park, USA, four small rodent species expanded distribution and nine rodent species contracted elevation ranges under a warming environment during 1914–2006 (Moritz et al., 2008). Approximately three‐quarters of studied small mammal species showed substantial elevational range shifts in montane California within a century (1911–2010) due to increase of air temperature (Rowe et al., 2015). In Europe, voles and lemmings showed a sustained population decline or collapse of population cycling (Gouveia et al., 2015; Kausrud et al., 2008; Prost et al., 2010; Selås et al., 2019), which may be related to climate warming and need to be investigated.

Anthropogenic disturbances are also thought to be a key driver for biodiversity loss and extinction of species (Young et al., 2016). A global analysis for 351 extinct mammal species shows that the increase of human population size was the major cause of mammal extinction in the past, and extinction rates of mammal species are predicted to increase with the increasing human populations in the future (Andermann et al., 2020). Human impacts such as hunting, land‐use change, livestock grazing, and human‐introduced species are shown to have a direct influence on mammal extinction (Smith et al., 2006; Young et al., 2016). For example, fossil evidence reflects that 67% of studied mammals have changed their climatic niches due to increased human activities (Pineda‐Munoz et al., 2021). Intensified and monoculture farming may have attributed to the population declines of European hamsters (*Cricetus cricetus*) in Europe (Kletty et al., 2019), and an increase of irrigation areas attributed to the population decline of an ever‐predominant rodent species, the Greater striped hamster (*Cricetulus barabensis*), in the northern plain of China (Yan et al., 2013).

Although climate warming and human disturbance are known to be closely associated with the range contraction of mammal species, quantitative assessments on their distinctive and interactive effects on local disappearance, as well as the underlying ecological processes, are still rare (but see: He et al., 2018; Wan et al., 2019; Wan & Zhang, 2017). Because human activities are often highly positively associated with climate warming in human history, it is necessary to distinguish their distinct effects on local extinction probability of species using spatial–temporal data, so as to take effective measures for wildlife management.

Brandt's voles (*Lasiopodomys brandtii*) are mainly distributed in the grasslands of China, Mongolia, and Russia (Avirmed et al., 2016). Brandt's voles are social small herbivores having burrow systems underground (Wang et al., 1992). They reproduce from April to August (Liu & Sun, 1993). Populations of Brandt's voles often fluctuate greatly across years driven by intrinsic and external factors with an interval about five years (Zhang et al., 2003). Brandt's voles used to be widely distributed in the steppe grassland of eastern Inner Mongolia in China, with its south range reaching the grassland in Hebei Province in China (Shi, 1988), but its southern distribution area experienced sharp shrinking over the past decades (Enkhbold et al., 2014), which may be attributed to significant reduction of genetic diversity of Brandt's voles (Li et al., 2017) and high levels of inbreeding (Wang et al., 2011). The historical distribution range of Brandt's voles and Mongolia gerbils (*Meriones unguiculatus*) widely overlapped in the Mongolian Plateau (Avirmed et al., 2016; Batsaikhan & Tsytsulina, 2016). The range contraction of Brandt's voles might make room for the population expansion of Mongolia gerbils, which are natural reservoirs of the plague bacterium *Yersina pestis* (Xu et al., 2015). A big irruption of plague caused by Mongolia gerbils occurred in Inner Mongolia during 2019–2020, resulting in a panic in November 2019 in Beijing, the capital of China, caused by arrival of two plague‐infected patients for treatments.

Accelerated climate warming and human activities may be responsible for the local disappearance or range contraction of their southern boundary of Brandt's voles in China, but this hypothesis has not been rigorously tested. Previous studies suggest that temperature may be a key factor limiting the distribution of Brandt's voles (Shi, 1988). The speed of climate warming in the study region is much faster than the average of the world (Qin, 2019). An increase of 0.27°C per decade of annual air temperature was reported in Inner Mongolia from 1960 to 2016 (Hao & Huang, 2019). Besides, Brandt's voles are sensitive to anthropogenic disturbances, such as cultivation and grazing, which also showed rapid increase in the grassland over the past decades. Intensive livestock grazing or cultivation may decrease the survival of Brandt's voles in the grasslands of Inner Mongolia (Li et al., 2016). The southern distribution range of Brandt's vole lies around the farming‐pastoral zone where human interference is more extensive (Zheng et al., 2014).

The purpose of this study aims to quantify the distinct associations of local disappearance of Brandt's voles with climate warming and anthropogenic disturbance by using historical records and by referring to the methods used for large mammals (Wan et al., 2019). During past decades, Brandt's voles have been well studied in China, so providing a good opportunity for evaluating the key factors attributing to local disappearance and range contraction of the voles, and for projecting its future change under accelerated climate warming, and then providing useful advice for better management of this species and the grassland ecosystem. In this study, we proposed two hypotheses: (a) The local disappearance probability of Brandt's vole should be positively associated with increased temperature, and southern populations would have experienced range contraction with more local disappearance and (b) the local disappearance probability of Brandt's vole should be positively associated with increased human population density.

## 2 MATERIALS AND METHODS

### 2.1 Species occurrence data

**FIGURE 1:**
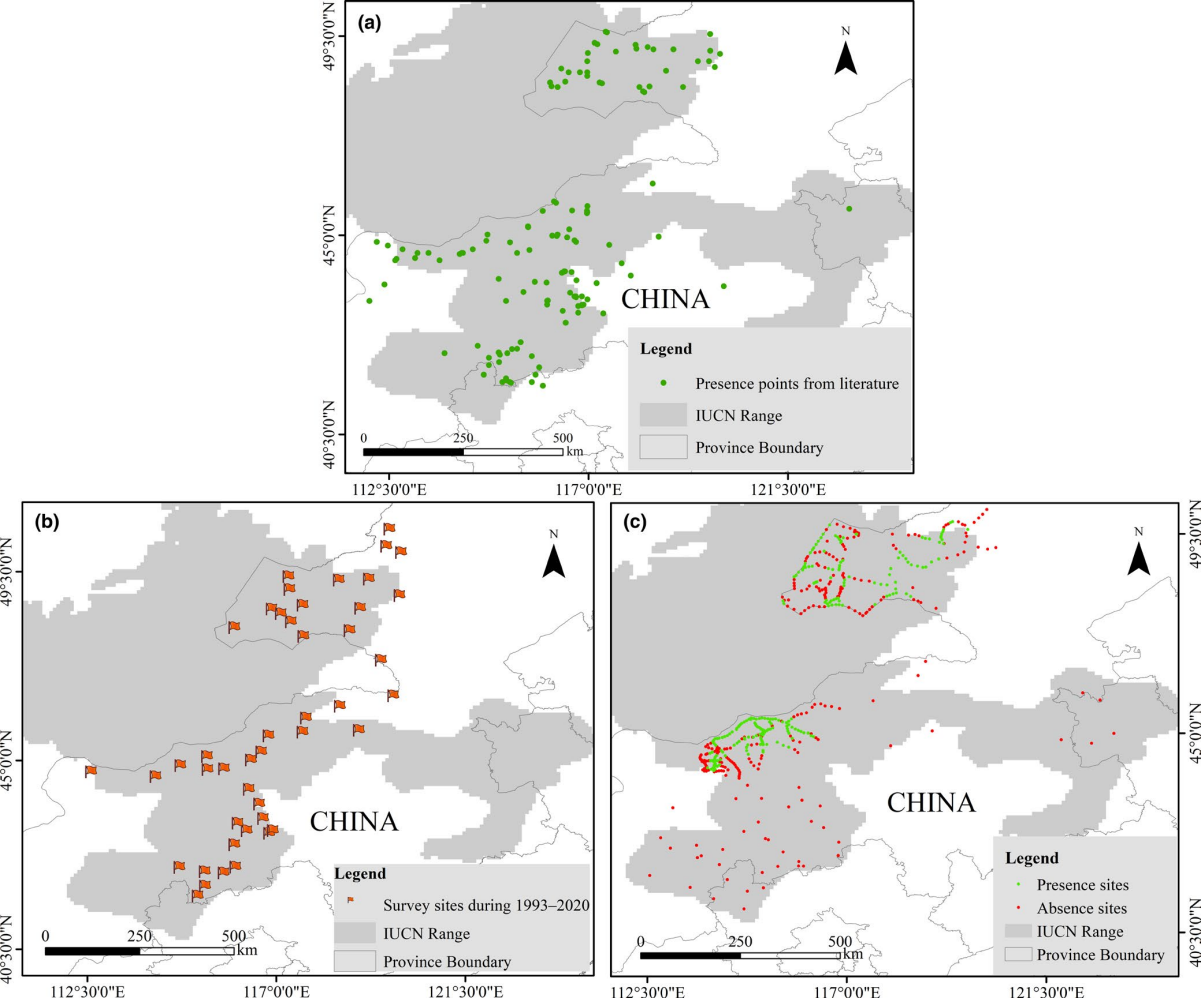
Presence points collected from literatures (a), regular field survey sites on rodent abundance during 1993–2020 (b), and Brandt's vole presence or absence verification investigation sites in 2019 (c) on the Inner Mongolia grassland. The grey colored background is recent IUCN ranges of Brandt's vole based on Red List of Threatened Species (Avirmed et al., 2016). And only 2 presence sites were collected from Global Biodiversity Information Facility (GBIF) and not displayed here

Every year from 1993 to 2020, we had regular field monitoring and surveys on rodent abundance in 45 locations on the Inner Mongolia grassland (Figure 1b; Table S7). To complement these data and add additional sites to our analysis, we collected all available occurrence data of Brandt's voles from 1970 to 2020 using several search engines. “Brandt’ vole,” “*Lasiopodomys brandtii*,” and “*Microtus brandti*” were used as key words in ISI Web of Knowledge (https://www.webofscience.com/) and Google Scholar (https://www.scholar.google.com). “*Bushi tianshu* (in Chinese),” “*Lasiopodomys brandtii*,” and “*Microtus brandti*” were used as key words in CNKI.net (http://www.cnki.net/). Online databases from Global Biodiversity Information Facility (GBIF; https://www.gbif.org/) and iNaturalist (https://www.inaturalist.org/) with occurrences records of Brandt's voles were also searched using “Brandt's vole” as key word. Because there is very little spatial–temporal distribution data from Mongolia and Russia, our study only focused on analysis of Brandt's voles in China (Table S1). To check for current presence or absence of Brandt's voles, most of these sites were revisited during a survey in September 2019 (Figure 1c). The investigation covered 493 survey sites using various signs of presence, including captured individuals, active burrows, and sightings of individual rodents. Details on field surveys are shown in Supporting information.

All locations with presence record of Brandt's voles were designated into grid‐cells of 10 × 10 km^2^ in Inner Mongolia, China. Records without clear spatial–temporal information of Brandt's voles were removed. For records with detail location description (e.g., village or ranch smaller than 10 × 10 km^2^), we import 10 × 10 km^2^ shapefile grid‐cells into LocaSpace Viewer (https://www.tuxingis.com) where the earth satellite map is accessible, then we searched location name on LocaSpace Viewer via Amap (https://m.amap.com) and found specific 10 × 10 km^2^ grid‐cell for each location, and coordinates of the center of each grid‐cell were used to represent these locations. Data were further screened for quality control by following Wan et al. (2019). Only records with specific year and spatial descriptions with specific longitude and latitude information (272/2623) or specific location name (village or pasture smaller than 10 × 10 km^2^) (304/2623), which could be designated into specific grid‐cells of 10 × 10 km^2^, were used (Table S2). The other records with a larger time resolution (decade or unclear) and spatial resolution (township, county, league, prefecture, country, or unclear) were excluded for statistical analysis (2047/2623) (Tables S1 and S2). A few records from city area (e.g., Hohhot and Changchun) with specific year and location name were excluded (2/304), as they were likely due to human introduction, incorrect records or typing errors.

**FIGURE 2:**
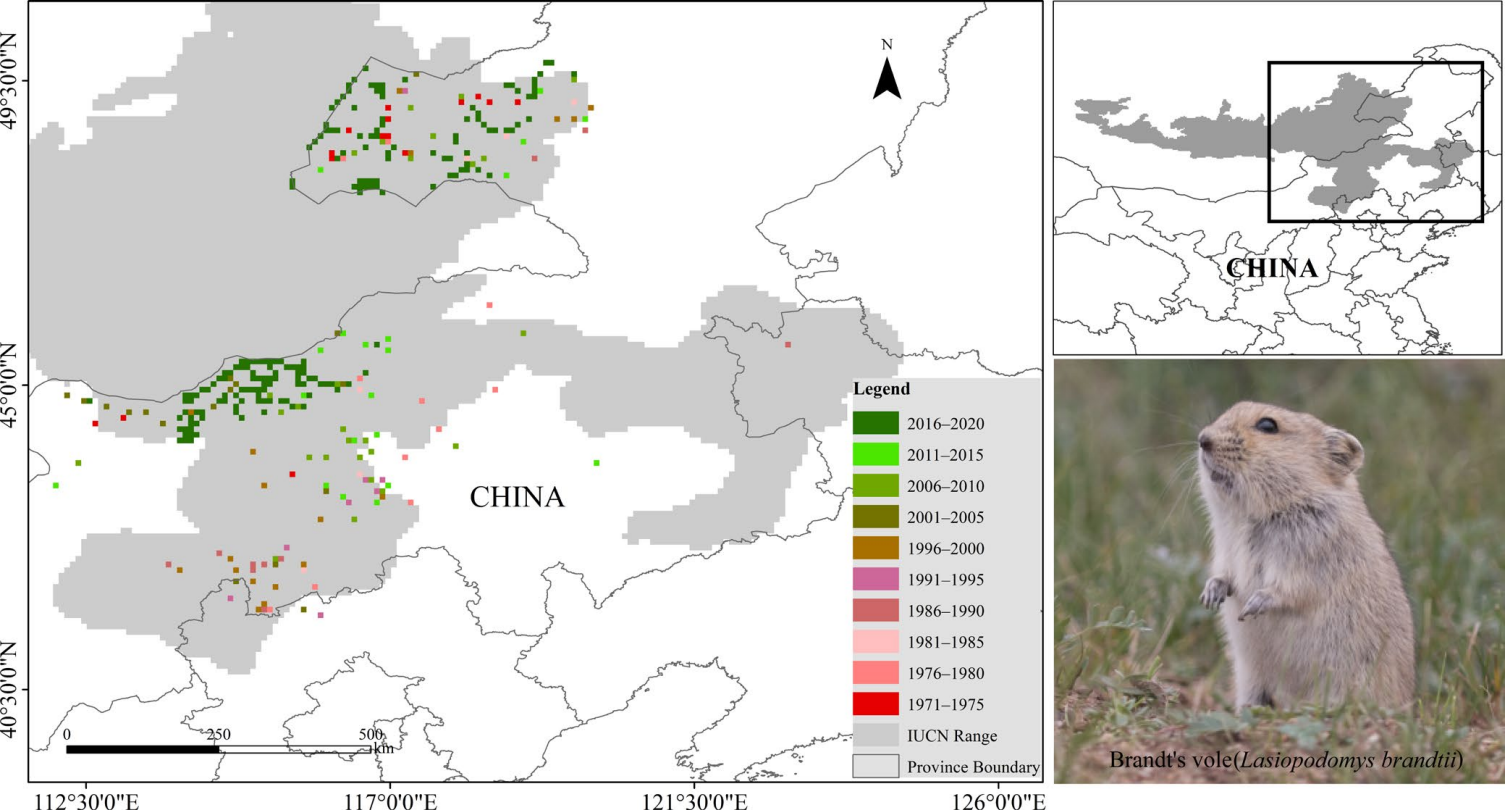
Last observation time of Brandt's voles in China during 1971–2020. The last observation time was defined as the year with last presence record of Brandt's voles in the grid‐cell (10 × 10 km^2^). The red and green colored grid‐cells represent earlier or later records of this study. The grey colored grid‐cells represent the distribution of Brandt's voles obtained from the IUCN Red List of Threatened Species (Avirmed et al., 2016)

By referring to Wan et al. (2019), the last recorded time (year) with presence of Brandt's voles in a grid‐cell was defined as the last observed time (year) (Figure 2). For the presence grid‐cells of Brandt's voles, if there was no presence record in literature for at least successive 5 years after the last observed time, and they were not found in the last extensive survey in 2019 and records in 2020, then these grid‐cells were defined as a local disappearance of Brandt's voles by following the definition of previous studies (Ávila et al., 2008; Steen, 1995). The current data and survey were not able to rule out the local extinction of voles in a grid‐cell, because if the density was too low to be detected in the 2019 survey. However, statistically, the disappearance probability could be used to reflect the range contraction of Brandt's voles, because if voles of all or most sites of its southern boundary disappeared, this means the species showed range contraction. Significant association of local disappearance probability with a climate or environmental variable suggests that the variable may attribute to range contraction of the Brandt's voles. Only grid‐cells with local disappearance of Brandt's voles were selected for further analysis. The yearly maximum air temperature (based on monthly values) of the year following the last observation year was used to estimate the disappearance threshold of Brandt's voles. We also calculated the disappearance threshold in the warmest month that may impose the critical threat to survival of voles in phases of climate warming. We determined the southern boundaries of Brandt's voles by linking grid‐cells with presence records located in the southern edge of their distribution from 1971 to 2020 with an interval of 5 years and then calculated the range shift by using the shortest distance between two grid‐cells of neighboring southern boundaries.

### 2.2 Climate and environmental variables

Historical climate data of temperature and precipitation from 1961 to 2018 were downloaded from WorldClim (https://www.worldclim.org). The yearly maximum air temperature was calculated by using the average values of monthly average maximum air temperature which are downscaled from CRU‐TS‐4.03 (Harris et al., 2014) using WorldClim 2.1 (Fick & Hijmans, 2017) for bias correction, and this was used to analyze the association between the local disappearance probability of voles and air temperature from 1971 to 2020. The yearly precipitation of each year was used to analyze the association between disappearance probability of voles and precipitation from 1971 to 2020. The normalized difference vegetation index (NDVI) obtained from the GIMMS NDVI3g dataset (https://iridl.ldeo.columbia.edu) had the longest time series (1981–2015) and it was used to represent changes in land use and vegetation. The NDVI dataset had a spatial resolution of 8 km and temporal resolution of half month (Pinzon & Tucker, 2014). Human population density (HPD, the number of persons per km^2^), which represent the anthropogenic stressors, was obtained from the History Database of the Global Environment (HYDE) (Goldewijk & Dr. ir. C. D. M, 2017). HYDE (3.2 2017 beta release) had a wide temporal scale and a spatial resolution of approximately 10 × 10 km^2^ (https://easy.dans.knaw.nl/ui/datasets/id/easy‐dataset:74467). Because we were interested in associations of local disappearance probability of Brandt's voles with changing climate and human disturbances, it was necessary to remove the regional difference of climate and environmental factors. Therefore, the yearly value (*Y_t_*) of air temperature, precipitation, NDVI, and HPD of each grid‐cell was standardized by the equation: (*Y_t_* − M)/SD, following Wan et al. (2019). M is an average value and SD is the standard deviation of *Y_t_* in a grid‐cell during the study period. Pearson correlation coefficient was used to test the relationship between these variables. The correlation among all independent variables is shown in Table S3. To avoid collinearity, we removed one of the variables if their correlation coefficient >0.5.

### 2.3 Statistical analyses

We used Pearson correlation coefficient to detect associations of range contraction as measured by the proportion of survived grid‐cells with climate and environmental variables (air temperature, precipitation, HPD, and NDVI).

**FIGURE 3:**
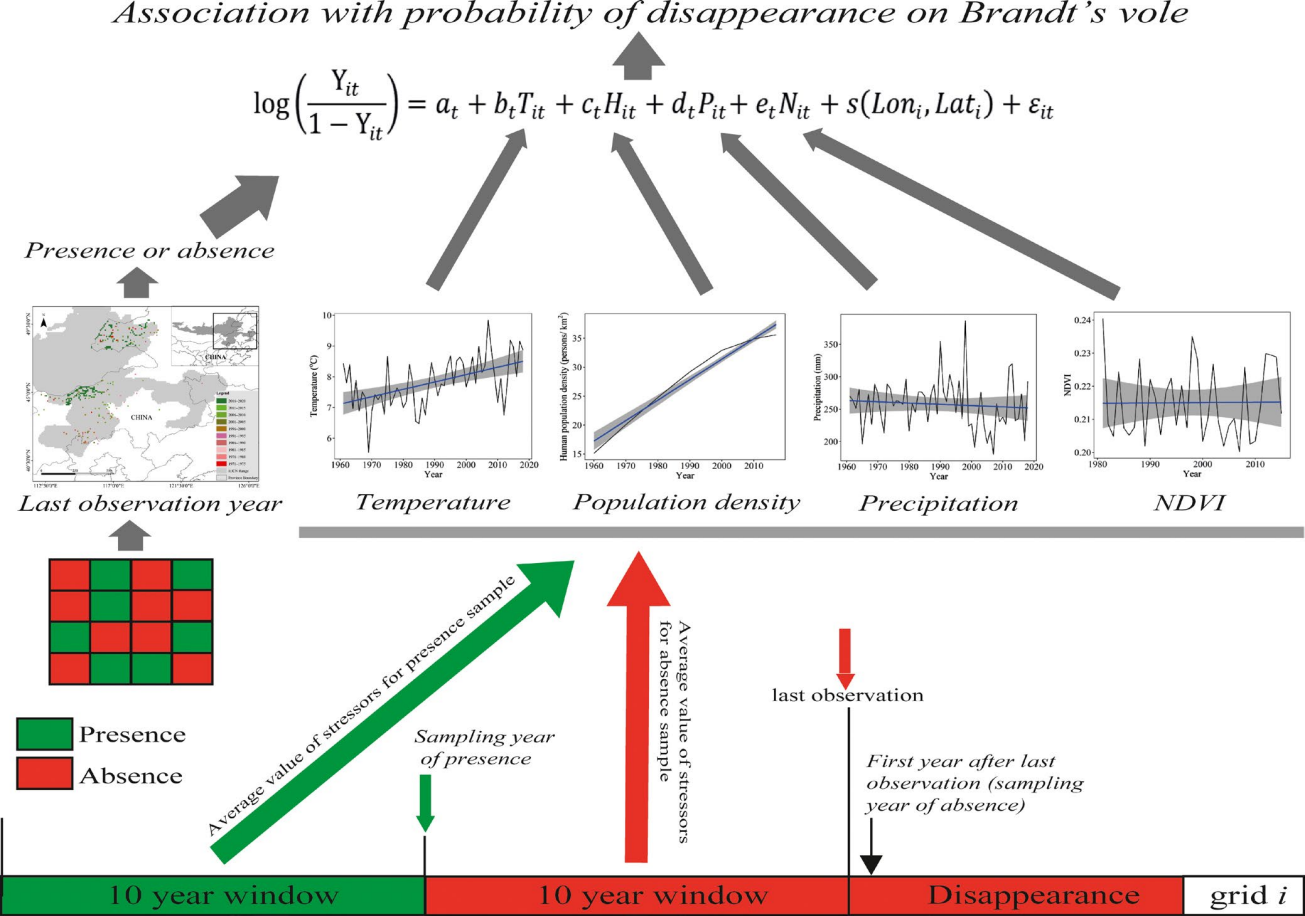
Diagram of the GAM method to calculate the associations of temperature, HPD, precipitation, and NDVI with probability of disappearance on Brandt's voles in Inner Mongolia, China. For each grid‐cell, there are a pair of data of local disappearance (0 representing survival, 1 representing disappearance) and associated climate and environmental variables. The absence year was defined as the year after the last observation year. The year that was 10 years before the absence year was selected as the presence year. The environmental variables (HPD, precipitation, NDVI, and air temperature) are the average value for the last successive 10 years before the presence or absence year. We compared associations of presence or absence of voles with their 10‐year average values of climate and environmental variables (by following Wan et al. (2019) with modifications). The last observation time map was consistent with Figure 2. Temperature, HPD, precipitation, and NDVI were consistent with Figure 4b, c, d, and e, respectively

To identify the effects of climate change and human disturbance on local disappearance probability, we compared associations of presence or absence of voles with climate and environmental variables by following Wan et al. (2019) (Figure 3). First, we choose the presence and absence year of voles. The absence year was defined as the year after the last observation year. The year that was 10 years before the absence year was selected as the presence year. The 10‐yr average values of environment variables before the presence and absence year were defined as the environmental values for presence and absence year, and they were used to infer the associations between presence or absence of voles with their 10‐yr average values of environmental variables. We assumed that Brandt's voles could have experienced sustained climate or human stressor of ten years (covering two cycles of population variation) before local disappearance. We used the average value of last ten years before the year of presence or absence of Brandt's voles which would cover two peak periods of the voles (Zhang et al., 2003). Thus, for each grid‐cell, there are two abundance data (presence or absence), two temperature data (average temperature of last ten years before the year of presence or absence), two precipitation data (average precipitation of last ten years before the year of presence or absence), two NDVI data (average NDVI of last ten years before the year of presence or absence), and two human density data (average human density of last ten years before the year of presence or absence). Then, with this information, each grid‐cell had a pair of data of local disappearance (0 representing survival, 1 representing disappearance) and associated climate and environmental variables. The environmental variables (HPD, precipitation, NDVI, and air temperature) are the average value for the last successive 10 years before the presence or absence year (Table S8).

A logistic generalized additive model (GAM) of the local disappearance probability (*Y_it_*) against the standardized temperature (*T_it_*), standardized HPD (*H_it_*), standardized precipitation (*P_it_*), and standardized NDVI (*N_it_*) was fitted following the formula:

(1) $$\log\left(\frac{Y_{\mathrm{it}}}{1‐Y_{\mathrm{it}}}\right)=a_{t}+b_{t}T_{\mathrm{it}}+c_{t}H_{\mathrm{it}}+d_{t}P_{\mathrm{it}}+e_{t}N_{\mathrm{it}}+s\left({\text{Lon}}_{i},{\text{Lat}}_{i}\right)+\varepsilon_{\mathrm{it}}$$

where *Y_it_* represented the dependent variable in the *i*th grid‐cell at time *t*. *T_it_*, *H_it_*, *N_it_*, and *P_it_* represent the standardized air temperature, standardized HPD, standardized NDVI, and standardized precipitation, respectively. Because correlations of these independent variables were not significant (Table S3), thus, all of them were included in the model analysis. *s*(Lon*_i_*, Lat*_i_*) is a 2D smoothing function (*k* value = 4) for modeling the spatial autocorrelation effects (Wan et al., 2019). *a_t_* is an intercept. *b_t_*, *c_t_*, *d_t_*, and *e_t_* represent the association of standardized temperature, standardized HPD, standardized NDVI, and standardized precipitation with the local disappearance probability of Brandt's vole. *ε_it_* represented the random error term. Interactions between environmental variables (*T_it_*:*H_it_*, *T_it_*:*N_it_*, *T_it_*:*P_it_*, *H_it_*:*N_it_*, *H_it_*:*P_it_*, *N_it_*:*P_it_*) were assessed and models were selected by smaller value of unbiased risk estimators (UBRE). Partial autocorrelation function (PACF) and residual semivariogram were used to detect residual temporal correlations and residual spatial correlations (Wan et al., 2019). The mgcv library (v 1.8‐33) in R software was used to run all generalized additive models.

To further confirm the GAM results, we compared the difference of the most recent accessible environment variables between presence and absence grid‐cells of Brandt's voles (air temperature in 2018; precipitation in 2018; human population density in 2017; NDVI in 2015), and difference of environment variables of presence grid‐cells during 2010–2018 and those of absence grid‐cells 10 years before absence year (the year after last observation year). Shaprio–Wilk test was used to test the normality of data (Shapiro & Wilk, 1965). We did Mann–Whitney *U* test to examine the difference between presence and absence grid‐cells because all data did not follow the normal distribution except for NDVI of absence grid‐cells in 2015.

In order to understand the impacts of future climate change on local disappearance of Brandt's voles after 2020, GAM was used to model the local disappearance probability under future climate warming scenarios. Maximum monthly temperature during 2021–2040 with Shared Socio‐economic Pathways 370 (SSP3‐7.0) from WorldClim (https://www.worldclim.org) was used as environmental variables. Global climate model MRI‐ESM2‐0 from CMIP6 was used for its better performance in main regions of China (Xiang et al., 2021). Current distribution grid‐cells of Brandt's vole (with records during 2016–2020) were used for projection analysis. All these selected ranges were divided into grid‐cells of 10 × 10 km^2^. Probability of disappearance by 2040 in these grid‐cells without presence records was projected based on GAM results and was represented in ArcGIS software (version 10.8).

## 3 RESULTS

### 3.1 Effects of climate and environmental variables on range contraction

**FIGURE 4:**
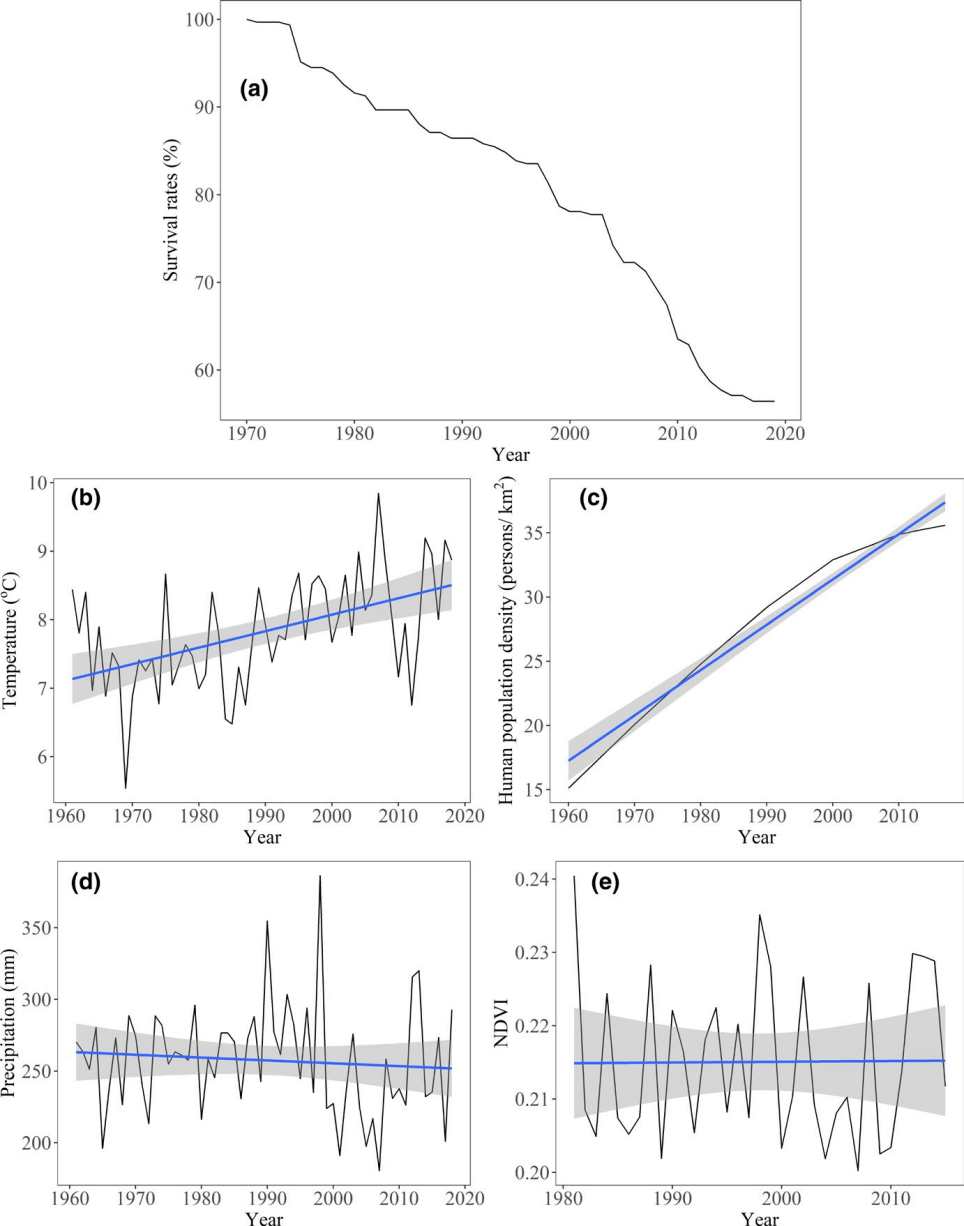
Temporal variation of proportion of survived grid‐cells of Brandt's voles (a), air temperature (b), HPD (c), precipitation (d), and NDVI (e) in China. Grey solid lines represent the temporal variation, and the blue lines represent their linear regression trend. The linear correlation with time for temperature and HPD are significant (all *p* < .001), while not significant for precipitation and NDVI (all *p* > .1). The grey shadow shows the 95% confidence interval of the regression

There was a total of 1150 historical presence records (534 from historical literatures, 40 from GBIF, 345 from field surveys during 1993–2020, and 231 from field surveys in 2019) of Brandt's voles assigned to 312 grid‐cells (10 × 10 km^2^) in China (Table S1, S2, S9; Figures 1 and 2). As compared to all historically distributed grid‐cells, the proportion of remaining grid‐cells showed a sharp decline with a reduction of approximately 50% from 1971 to 2019 (Figure 4a). Air temperature during 1961–2018 and HPD from 1960 to 2017 in the study area showed obvious increases (about 1.5°C for temperature and 20 persons per km^2^ for humans) (Figure 4b, C, all *p* < .001), while both precipitation during 1961–2018 (Figure 4d) and NDVI from 1981 to 2015 (Figure 4e) showed a nonsignificant linear trend (all *p* > .05).

Both temperature (*n* = 49) and HPD (*n* = 48) showed significant and negative correlations with the proportion of remaining grid‐cells of Brandt's voles from 1971 to 2019 (all *p* < .05), while both precipitation (*n* = 49) and NDVI (*n* = 35) showed a nonsignificant correlation (all *p* > .05, Table S4).

### 3.2 Effects of climate and environmental variables on local disappearance probability

Based on results of the best‐fitted spatial–temporal generalized additive model analysis without interaction effect, local disappearance probability of Brandt's voles showed a significant and positive association with temperature (*p* < .001) (Table 1; Figure S1A; Table S6). Based on results including interactive effect, temperature showed a significant positive association with local disappearance probability of Brandt's voles (Table 1; Figure S2A; Table S6); HPD showed a significant negative association with local disappearance probability of Brandt's voles (Table 1; Figure S2C; Table S6); HPD and precipitation showed a significant and positive interaction effect, precipitation and NDVI showed a significant and positive interaction effect, while temperature and precipitation showed a significant and negative interaction effect on local disappearance probability of Brandt's voles (Table 1). Residual temporal autocorrelation diagnostics showed that partial autocorrelation coefficients gradually converge with the increase of lag number (Figure S4A,B). All residual temporal autocorrelation diagnostics results showed first‐order temporal autocorrelations (Figure S4A,B).

**TABLE 1:** Associations of standardized temperature, standardized precipitation, standardized HPD, and standardized NDVI with the local disappearance probability of Brandt's voles with and without interactive effects

Effect	Intercept	Standardized HPD	Standardized precipitation	Standardized temperature	Standardized NDVI	Standardized HPD: Standardized precipitation	Standardized HPD: Standardized temperature	Standardized precipitation: Standardized temperature	Standardized precipitation: Standardized NDVI	*s*(Latitude, Longitude, *k* = 4)
Without interactive effect	–0.41	–0.54	–1.22	2.96***	–0.78	–	–	–	–	6.59
With interactive effect	–0.86*	–1.08*	–2.14	3.99***	0.03	3.27**	–1.62	–6.19*	7.04**	13.63**

Note

Associations are represented by the regression coefficients using the best GAM model selected. Standardized HPD: Standardized precipitation, Standardized HPD: Standardized temperature, Standardized precipitation: Standardized temperature, and Standardized precipitation: Standardized NDVI represents their interactions. *s*(Latitude, Longitude, *k* = 4) represents the spatial autocorrelation effects.

*p < .05, **p < .01, ***p < .001.

The disappearance threshold of yearly maximum air temperature (i.e., average value of monthly maximum air temperature of a year) was estimated to be 8.18 ± 1.86°C (Figure S3A; Table S5). The average maximum air temperature of the warmest months in the first absence year among all grid‐cells was 27.50 ± 1.61°C (Figure S3B; Table S5).

The southern boundaries of Brandt's vole shifted rapidly from its south to north nearby the border between China and Mongolia (Figure 2). It was estimated that the southern boundary moved toward north pole with an approximate 287 km northward range shift from 1992 to 2020 based on the last observation time of southern boundary, corresponding to a temperature rise of approximately 1°C during 1992–2020 (or 0.36°C per decade) in the Inner Mongolia grassland.

Using Mann–Whitney *U* test, we found Brandt's voles in the absence grid‐cells faced greater environment pressure (e.g., higher temperature and high human population density) during last 10 years than that of presence grid‐cells (Figure S5), which support our GAM results using only absence data.

### 3.3 Projections of range shift

**FIGURE 5:**
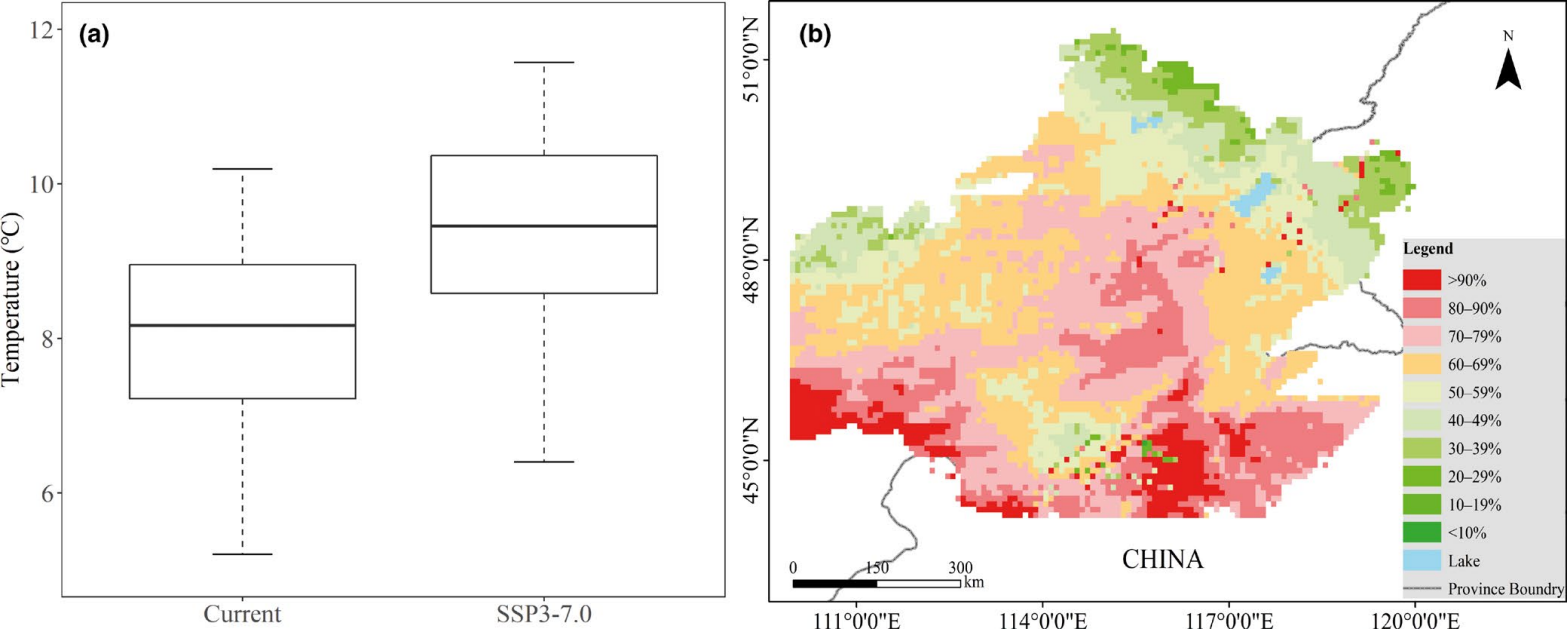
Projected local disappearance probability of Brandt's voles in China under warming conditions of Shared Socio‐economic Pathways 370 (SSP3‐7.0) by 2040 using GAM models without interaction effects. The distribution range of Brandt's voles during 2016–2020 based on IUCN Red List of Threatened Species (Avirmed et al., 2016) was used for projections of their future distribution. The annual mean temperature with current (2001–2018) and SSP3‐7.0 (2021–2040) conditions were calculated and plotted (a) for all projected 10 × 10 km^2^ grid‐cells (b)

GAM models showed that the local disappearance probability of Brandt's vole would increase and the range contraction of the southern boundary of Brandt's vole would continue under climate warming scenarios from 2021 to 2040 with Shared Socio‐economic Pathways 370 (SSP3‐7.0), which is equivalent to a rise of 1.3°C (Figure 5).

## 4 DISCUSSION

By using historical records, we found the spatial distribution of Brandt's voles in China experienced a dramatic range contraction from 1971 to 2020. The southern boundary of voles has retreated northward of 287 km from 1992 to 2020, close to the China–Mongolia border (Figure 2). We found climate warming was positively associated with local disappearance probability of Brandt's voles, particularly under years of drought. Human disturbance showed a negative association with local disappearance probability of Brandt's voles, but such association was positive in years with abundant precipitation. High vegetation coverage increased local disappearance probability in years with abundant precipitation. Our results support the first hypothesis, but not support the second hypothesis except for the case of interaction with precipitation. The disappearance threshold of yearly air maximum temperature was estimated to be 8.42 ± 1.89°C (27.50 ± 1.61°C in the warmest month). Based on our projections under climate warming scenarios from 2021 to 2040, range contraction and local disappearance probability of Brandt's voles would be further accelerated in their southern boundaries. Our study highlights the necessity of identifying the distinctive and interactive effects of climate change and human disturbances on species disappearance and range shift or contraction during the past decades for a better management of wildlife. Our findings provide novel insights into the ecological process of local disappearance and range contraction of Brandt's voles on the steppe grassland, and implications for a better management of this keystone species in the grassland ecosystem under accelerated climate change and human disturbance.

### 4.1 Impacts of climate warming

Recently, climate warming has been suggested to be associated with range contraction of many small mammal species (Rowe & Terry, 2014). However, the ecological process of range contraction has rarely been investigated quantitatively, particularly by excluding the correlative effects of human disturbances. In this study, we found climate warming significantly increased the local disappearance probability of Brandt's voles from 1971 to 2020, resulting in range contraction of their southern boundary, supporting our first hypothesis.

There are two plausible explanations for the observed effects of climate warming on voles in the Inner Mongolia grassland. First, the rise of temperature may significantly disrupt the physiological functions of Brandt's voles. Previous studies found high temperature impaired spermatogenesis in testis of rats (Kanter et al., 2013). Reed voles regulated their reproduction time and decreased litter size in response to warm temperatures (Guo et al., 1999). Milk production and pup growth of common voles (*Microtus arvalis*) reduced in a warmer environment (Simons et al., 2011). And common voles reorganized their behaviors associated with lactation in adaptation to a warm environment (Vincent et al., 2014). Extreme high temperature was found to elevate metabolic rates and decrease cognitive abilities for rats (Schmit et al., 2017). High temperature may reduce reproductive success or increase the risk of disease transmission in Brandt's voles. A recent laboratory study showed that higher temperatures that lasted for 27 days at 32°C could decrease immunity in Brandt's voles (Xu et al., 2019). In addition, gut microbiota was connected with the thermoregulation of rodents in response to temperature changes (Khakisahneh et al., 2020). The frequency of extreme temperature events significantly increased in Inner Mongolia of China during 1960–2017 (Tong et al., 2019), and high frequency of physiological responses to high temperature events may reduce the survival rates of the Brandt's vole. The average temperature of its southern boundary is relatively higher than the northern part and this increase in temperature well explains why voles disappeared much earlier in the south than in the north. In this study, we were able to estimate the disappearance threshold of yearly maximum air temperature (8.42 ± 1.89°C) and the average maximum temperature of the warmest months (27.50 ± 1.61°C) at which there was evidence of local vole disappearance. And the estimated disappearance threshold of the warmest months was very similar to the lower critical temperature (27.5°C) of the thermal neutral zone of Brandt's voles (Li & Huang, 1994). When air temperature is greater than the upper critical thermal limits, voles face a high probability of disappearance (Bennett et al., 2021). In 2004, population translocation of Brandt's voles (*n* = 200) from Ehebaolige in Dongwuqi (a northern distribution area) to Jilinwusu in Taipusiqi (the historical southern boundary and a long‐term research site for Brandt's voles) was conducted, and no voles survived into the next year (Dazhao Shi, unpublished), suggesting the historical site is not suitable for voles to live.

Second, climate warming may cause local disappearance through altering plant composition. Climate warming might favor plant invasion into the semi‐arid grassland (Blumenthal et al., 2016). Warming was found to firstly remove perennials and Cyperaceae species over annuals in a tallgrass prairie (Craine et al., 2011). Brandt's vole is an herbivorous mammal that prefers *Leymus chinensis* and *Stipa capillata*, which are perennials on the Inner Mongolia grassland (Wang et al., 1992). An increase in temperature may reduce their favorite plant species. Warming was also found to decrease the productivity of plants in grasslands (Boeck et al., 2007). On the Inner Mongolia grassland, climate warming caused significant grassland degradation (Ma et al., 2017), which may impose a threat to Brandt's voles. Changes in grassland vegetation community attributes could reduce the quantity and quality of preferred vegetation species of Brandt's voles, might have profound impacts on the survival of offspring, and on their body regulation ability (Lou, 2013). In addition, the change of food resource composition for the Brandt's vole might contribute to the local disappearance due to a narrower niche and less food choices compared with sympatric rodents (Yue et al., 2020). Recently, researchers reported that increased precipitation was helpful for the recovery of *Leymus chinensis* and altered the gut microbiome through changed food composition (Li et al., 2020). The rise of temperature may also change the gut microbes of Brandt's voles through changing food ingredients, thereby affecting their growth.

### 4.2 Impacts of precipitation

There is solid evidence that abundant precipitation would increase the population of rodents by increasing food resources in various ecosystems in North America (Brown & Ernest, 2002), Australia (Singleton & Redhead, 1990), western South America (Lima et al., 1999), China (Li & Zhang, 1993), and Africa (Leirs, 1999). For example, abundant precipitation increased population abundance of *Peromyscus leucopus* by increasing its food resources in North America (Brown & Ernest, 2002). In southern and eastern Australia, high rainfall triggered outbreaks of house mice (*Mus muscuslus domesticus*) after prolonged droughts (Singleton & Redhead, 1990). In Africa, high rainfall caused an eruption of an African rat (*Mastomys natalensis*) population due to the increase in food resources (Leirs, 1999). Previous studies suggest that abundant precipitation also facilitates population increases in Brandt's voles on the Inner Mongolia grassland (Zhang et al., 2003). In this study, we found the local disappearance probability of Brandt's voles was significantly and positively associated with the decrease of NDVI when precipitation was low. Drought often causes desertification of the grasslands, which is detrimental to voles due to lack of food resources. This may explain why drought may increase the local disappearance probability of Brandt's voles, particularly under warming conditions.

### 4.3 Impacts of human disturbances

A global meta‐analysis of the literature showed that human disturbances caused nearly a one‐fifth decline of species richness on our planet (Murphy & Romanuk, 2014). Although small mammals are often more resistant to human disturbances than large ones, there is growing evidence that some small rodent species are facing range contraction under accelerated human pressures. For example, maize monoculture caused the local extinction of the common hamster (*Cricetus cricetus*) in Western Europe from 1937 to 2014 (Tissier et al., 2017). Extensive ploughing activities in central Germany from 1998 to 1999 significantly suppressed populations of the common vole (*Microtus arvalis*) (Jacob, 2003). It is notable that during the past century, the increase in human activities is highly associated with climate warming. Thus, it is necessary to separate their distinctive effects of climate change. In this study, we found HPD was significantly and negatively associated with the local disappearance probability of Brandt's voles using model with interactive effects (not supporting the second hypothesis), and the local disappearance probability of Brandt's voles was significantly and positively associated with the increase of HPD when precipitation was high (partially supporting the second hypothesis).

Brandt's voles are social small rodents and are quite resistant to disturbances to the grassland. They prefer moderately disturbed and open habitats for easily sensing predators (Zhong et al., 1999). Previous research found population density of Brandt's voles was positively associated with grazing activities (Ren et al., 2011), which may explain why HPD had a negative effect on local disappearance probability. However, over‐grazing may have negative effects on rodent populations, and manipulation studies in large‐scale enclosures have demonstrated that successive grazing significantly reduced the population abundance of Brandt's voles due to a shortage of food resources (Li et al., 2016). Grazing prohibition and grassland restoration programs in Inner Mongolia were launched over 20 years ago (Chen, 2020), which reduced the overdegradation of the grassland. However, the grassland is being converted into farmlands in some places where precipitation is high. In years with abundant precipitation, there may be more cultivation and this decreases the probability of survival.

### 4.4 Speed of range shift

Range shifts were observed in many taxonomic groups under climate warming conditions (Chen et al., 2011). A global meta‐analysis documented significant range shifts averaging 6.1 km per decade (about 0.1°C increase in temperature) toward the poles (Parmesan & Yohe, 2003). Chen et al. (2011) estimated that species shifted to higher latitudes at rate of 16.9 km per decade (about 0.1°C increase of temperature). Wan and Zhang (2017) predicted large mammals might move approximately 150 to 200 km toward the north with 1°C increase in temperature. Our study found that the southern boundary of Brandt's voles was shifted to higher latitudes by at least 287 km between 1992 and 2020 in China (about 0.36°C increase of temperature per decade). The moving speed of Brandt's voles was about two times faster than that of previous studies, indicating they are particularly sensitive to climate warming.

### 4.5 Limitation of this study

The study would suffer limitation of estimating the local disappearance probability because many grid‐cells were not continuously surveyed using literature survey. For some grid‐cells with fewer previous surveys, the local disappearance probability depending on 2019–2020 survey would be overestimated. However, statistically, this estimation was still useful because over 100 absence sites (Table S8) were used for analysis which could help to minimize the biased estimations. Future studies could apply an approach that randomly samples the disappearance data between last observed presence and observed absence as to account for stochasticity by averaging the GAM results across these replicates. Despite these defects, our approach provides an opportunity of estimating range contraction of animals using incomplete occurrence data in historical literatures.

### 4.6 Implications for management

Population density of the Brandt's vole changes greatly in different years, driven by both extrinsic and intrinsic climate factors (Zhang et al., 2003). In outbreak years, the population density of Brandt's voles can reach 500–800 voles per ha, which caused great damage to grassland and to poultry production (Wan et al., 1988). Therefore, before 1990s, Brandt's voles were often taken as a significant rodent pest species. The range contraction in the southern part of the Inner Mongolian grassland significantly eased the damage the rodents caused, but may have also disrupted the normal ecological function and service of the steppe grassland ecosystem. Brandt's vole is one of the most abundant small rodent species in the steppe grassland of Inner Mongolia (Li et al., 2017). Brandt's voles are important prey for many local predators (Gombobaatar et al., 2012). The burrowing activities of rodents play a significant role in maintaining biodiversity of the ecosystem (Davidson et al., 2012). Thus, regional disappearance of Brandt's voles may impose a threat to biodiversity conservation in the steppe grassland, and this possibility needs further monitoring and research. It is necessary to conserve the patchy populations of Brandt's voles where regional disappearance of voles was observed.

Another problem of regional disappearance of Brandt's voles is the worsening invasion or expansion of populations of Mongolian gerbils, which may carry more lethal plague pathogen (*Yesenia pestis*) in the Inner Mongolia grassland. Mongolia gerbils prefer desert or degraded habitats, while Brandt's voles prefer the steppe grassland. The plague pathogen carried by Brandt's voles is less infectious to people as compared to those of Mongolia gerbils. The regional disappearance of Brandt's voles facilitated the population expansion or invasion of Mongolia gerbils, posing a threat to human health. The underlying mechanisms mediating the interspecific competition of the two sympatric species under climate warming need further investigation.

## CONFLICT OF INTEREST

All authors declare no conflict of interests.

## AUTHOR CONTRIBUTION

**Defeng Bai:** Data curation (equal); Formal analysis (lead); Writing – original draft (lead); Writing – review & editing (lead). **Xinru Wan:** Data curation (equal); Formal analysis (lead); Methodology (equal); Writing – original draft (lead); Writing – review & editing (equal). **Guoliang Li:** Data curation (equal); Investigation (equal). **Xinrong Wan:** Data curation (equal); Investigation (equal). **Yongwang Guo:** Data curation (equal); Writing – review & editing (equal). **Dazhao Shi:** Writing – review & editing (equal). **Zhibin Zhang:** Conceptualization (lead); Data curation (equal); Formal analysis (equal); Funding acquisition (lead); Methodology (equal); Project administration (lead); Writing – original draft (equal); Writing – review & editing (lead).

## Supporting information

Supplementary Material

Table S9

## Data Availability

The data that support the findings of this study are available in the supporting information of this article.
