# Supplementary material for: Factors influencing range contraction of a rodent herbivore in a steppe grassland over the past decades
Source: Ecol Evol. 2022 Feb 14;12(2):e8546. doi: 10.1002/ece3.8546 (PMC8843818; doi:10.1002/ece3.8546)
Supplement: Supplementary file 1 — Supplementary Material [file ECE3-12-e8546-s001.docx]

**Supplementary Information for**

**Factors influencing range contraction of a rodent herbivore in a steppe grassland over the past decades**

Defeng Bai^1,2^ | Xinru Wan^1,2^ | Guoliang Li^1,2^ | Xinrong Wan^1^ | Yongwang Guo^3^ | Dazhao Shi^4^ | Zhibin Zhang^1,2^

**Correspondence**

Zhibin Zhang

Email: [zhangzb@ioz.ac.cn](mailto:zhangzb@ioz.ac.cn)

**This file includes:**

Methods

Table S1 to S9

Figure S1 to S5

References for SI reference citations

Data table (Table S7, Table S8 and Table S9)

**Methods**

**Data collection**

For collecting published online documents with distribution data of Brandt’s voles, ISI Web of Knowledge (<https://www.webofscience.com/>), Google Scholar (https://[www.scholar.google.com](http://www.scholar.google.com)) and CNKI.net (http://www.cnki.net/) were selected as search engines. “Brandt’s vole”, “*Lasiopodomys brandtii*” and “*Microtus brandti*” were used as key words in ISI Web of Knowledge and Google Scholar. “*Bushi tianshu* (in Chinese)”, “*Lasiopodomys brandtii*” and “*Microtus brandti*” were used as key words in CNKI.net where comprehensive Chinese literatures (Academic journal papers, Master’s and PhD thesis in Chinese) about Brandt’s voles can be obtained. We browsed and filtered out literatures with distribution information (when and where voles occurred) of Brandt’s voles. Occurrence time and spatial information of Brandt’s voles were recorded for further processing. In addition, online databases from Global Biodiversity Information Facility (GBIF; https://www.gbif.org) and iNaturalist (https://www.inaturalist.org/) with occurrences records of Brandt’s voles were also searched using “Brandt’s vole” as key word (Table S1).

In order to survey the status and population changes of rodent species in Inner Mongolia, 45 sites covering the northern and southern distribution range of Brandt’s voles in China had been selected for conducting regular field monitoring and surveys on rodent abundance on the Inner Mongolia grassland during 1993–2020 (Figure 4B; Table S6). Live traps or clips were used to catch Brandt’s voles. The longitude and latitude of each site was recorded, and the presence (0) or absence (1) status of Brandt’s vole was recorded for sites surveyed each year. And in order to reconfirm the current distribution status of Brandt’s voles in its southern range, a field investigation was carried out in September 2019 (Figure 4C). The investigation covered 493 survey sites using various signs of presence, including captured individuals, active burrows, and sightings of individual rodents. The distance between two sites was at least 1 km. When one site was selected, we recorded the coordinates and looking for traces of Brandt’s voles (active burrows and sightings of Brandt’s voles), we set up live traps in sites with high frequency of activities (more fresh traces) for capturing and sampling. Burrows of Brandt’s voles are easy to distinguish from sympatric rodent species like Daurian ground squirrels (*Spermophilus dauricus*) and Mongolia gerbils (*Meriones unguiculatus*) (Shi, 1985; Li et al., 1988; Gao & Wang, 2012). We recorded the status as “presence” as long as one type of trace (captured individuals, active burrows, and sighting of individuals) was found. The presence (0) or absence (1) status of Brandt’s vole was recorded for each site.

For all presence records, time resolutions were classified into three levels (specific year records, records not designated to specific year but with one decade, and unclear records without time information) and spatial resolutions were classified into eight levels (specific location with latitude and longitude recorded, specific location description which can be designated into specific grid-cells of 10 × 10 km^2^, presence information with only township description, presence information with only county description, presence information with only league description, presence information with only prefecture description, presence information with only country description, and presence records without spatial information). For records with detail location description (e.g., village or ranch), we import 10 × 10 km^2^ shapefile grid-cells into LocaSpace Viewer (https://[www.tuxingis.com](http://www.tuxingis.com)) where the earth satellite map is accessible, then we search location name on LocaSpace Viewer via Amap (<https://m.amap.com>) and find specific 10 × 10 km^2^ grid-cell for each location, and coordinates of the center of each grid-cell were used to represent these locations. Amap (supported by AutoNavi Software Co., Ltd. of Alibaba Group) is the largest provider of mobile digital map in China and updates very instantly to provide navigation and ride-hailing services.

Due to the lack of occurrence records in Mongolia and Russia, we only used presence records of Brandt’s voles in China for statistical analysis. Records without clear spatial-temporal descriptions of Brandt’s voles were removed. Only records with both high-resolution place and time information were used for analysis. Records with high-resolution places (specific location with latitude and longitude recorded or specific location description which can be designated into specific grid-cells of 10 × 10 km^2^) were regarded as clear spatial descriptions. Other records with low spatial resolution (presence information with only township description, presence information with only county description, presence information with only league description, presence information with only prefecture description, presence information with only country description, and presence records without spatial information) were excluded for statistical analysis (Table S2). Records with high-resolution time included specific year. Other records with decade or unclear time resolution were excluded for statistical analysis (Table S2).

**Reference**

Avirmed, D., Batsaikhan, N., & Tinnin, D. (2016). *Lasiopodomys brandtii*. The IUCN Red List of Threatened Species, e.T11340A115101423. <https://dx.doi.org/10.2305/IUCN.UK.2016-3.RLTS.T11340A22351917.en>

Gao, G., & Wang, S. W. (2012). Plague host animals and their control in China. Gansu Science and Technology Press, Lanzhou.

Li, Z. Q. (1988). Identification of Daurian ground squirrels burrow system and analysis of its status. Chinese Journal of Control of Endemic Diseases, 5, 314.

Shi, D. Z. (1985). A preliminary study on the distribution pattern of mousehole in Brandt’s voles. Journal of Inner Mongolia College of Agriculture & Animal Husbandry, 6(2), 111–117.

Wan, X. R., Jiang, G. S., Yan, C., He, F. L., Wen, R. S., Gu, J. Y., Li, X., Ma, J., Stenseth, N. C., & Zhang, Z. B. (2019). Historical records reveal the distinctive associations of human disturbance and extreme climate change with local extinction of mammals. Proceedings of the National Academy of the Sciences of the United States of America, 116(38), 19001–19008. <https://doi.org/10.1073/pnas.1818019116>


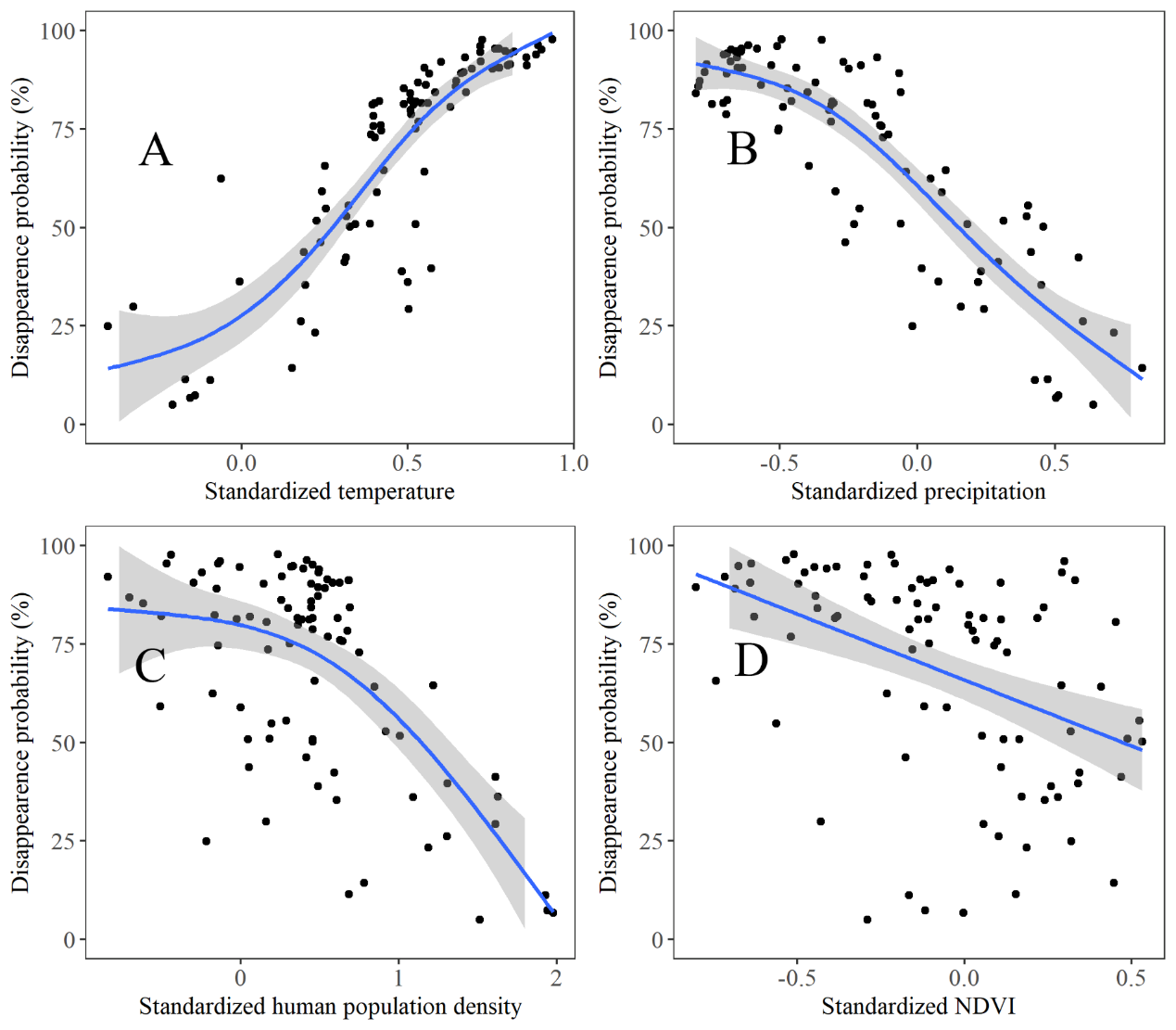


FIGURE S1 Relationship of the local disappearance probability of Brandt’s voles with the standardized temperature (A), standardized precipitation (B), standardized human population density (C) and standardized NDVI (D) by using GAM models without interactive effects. Black solid dots represent the local disappearance probability for each grid-cell. Blue lines represent the logistic regressions (*p* < 0.01 for A, *p* > 0.05 for B, C and D, the confidence interval is 95%). Temperature showed significant positive effect on the local disappearance probability of Brandt’s voles. Precipitation, human population density and NDVI showed non-significant negative effects on the local disappearance probability of Brandt’s voles.


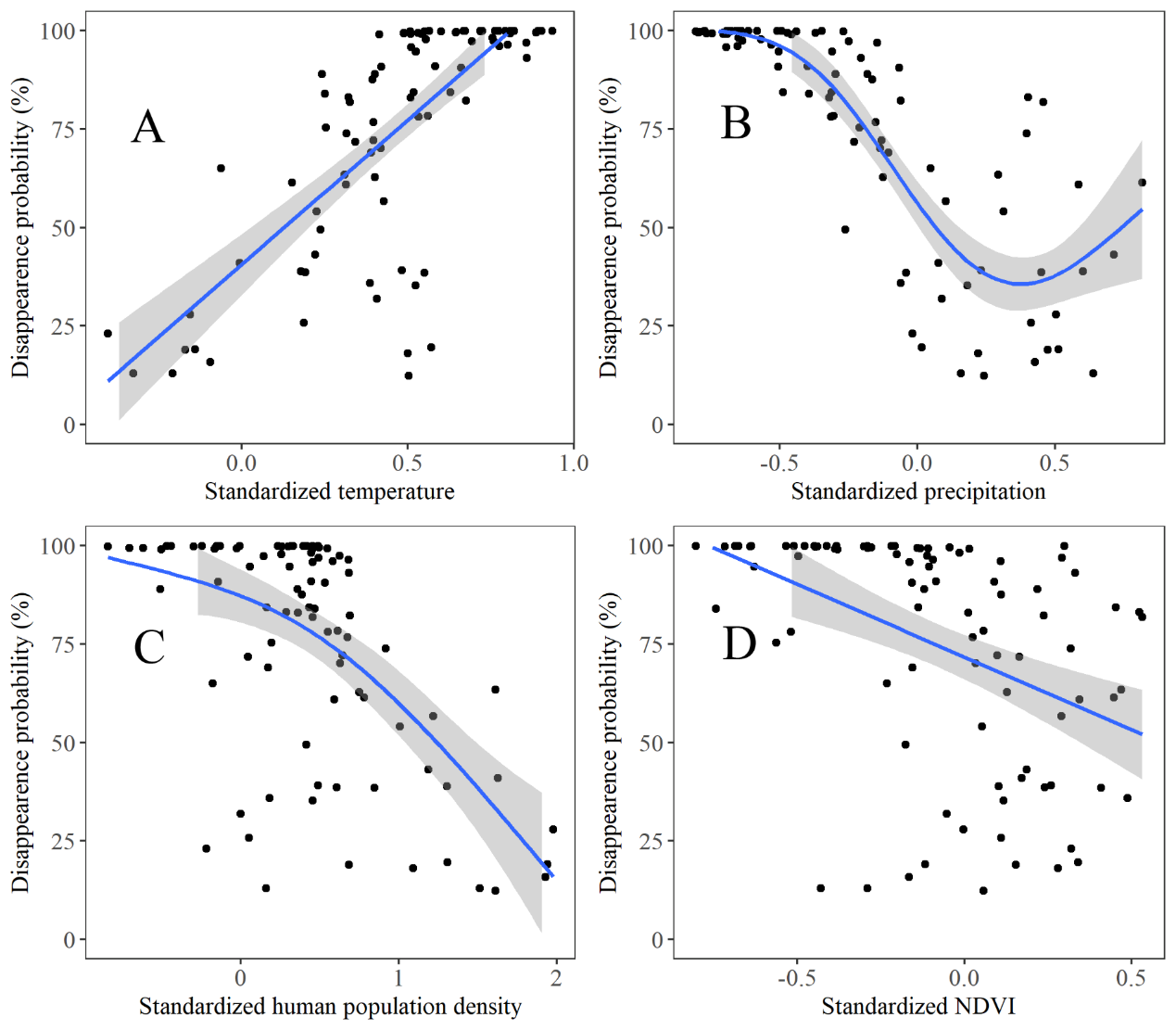


FIGURE S2 Relationship of the local disappearance probability of Brandt’s voles with the standardized temperature (A), standardized precipitation (B), standardized human population density (C) and standardized NDVI (D) by using GAM models with interactive effects. Black solid dots represent the local disappearance probability for each grid-cell. Blue lines represent the logistic regressions (*p* < 0.05 for A, *p* > 0.05 for B, C and D, the confidence interval is 95%). Temperature showed significant positive effect on the local disappearance probability of Brandt’s voles. Human population density showed significant negative effect on the local disappearance probability of Brandt’s voles. Precipitation and NDVI showed non-significant negative effects on the local disappearance probability of Brandt’s voles.


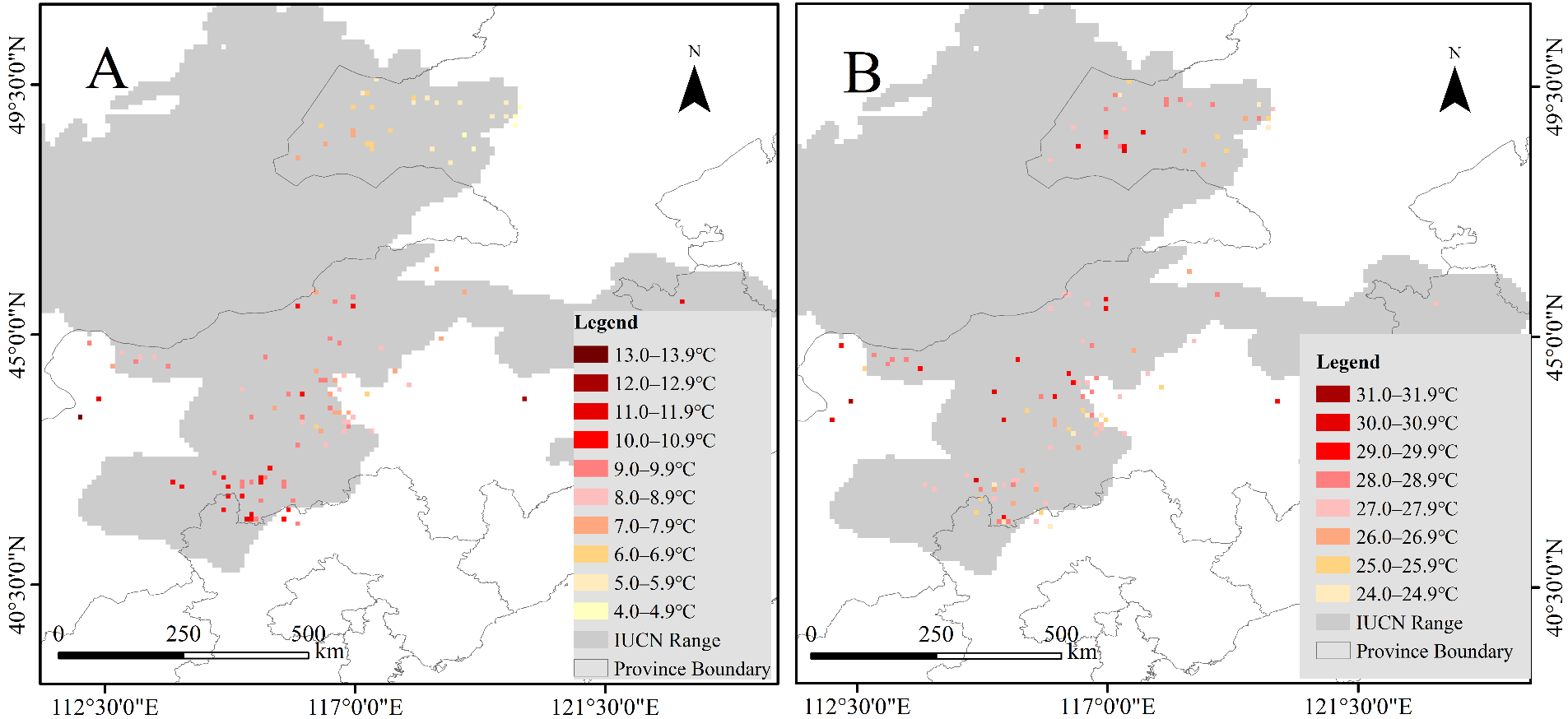
FIGURE S3 Yearly maximum air temperature (A) and maximum air temperature of the warmest month (July) (B) in the disappearance (absence) year of Brandt’s voles in each grid-cell. The grey colored background is the distribution of Brandt’s voles based on Red List of Threatened Species (Avirmed et al., 2016). The darker red color indicates higher temperature in the first disappearance (absence) year.


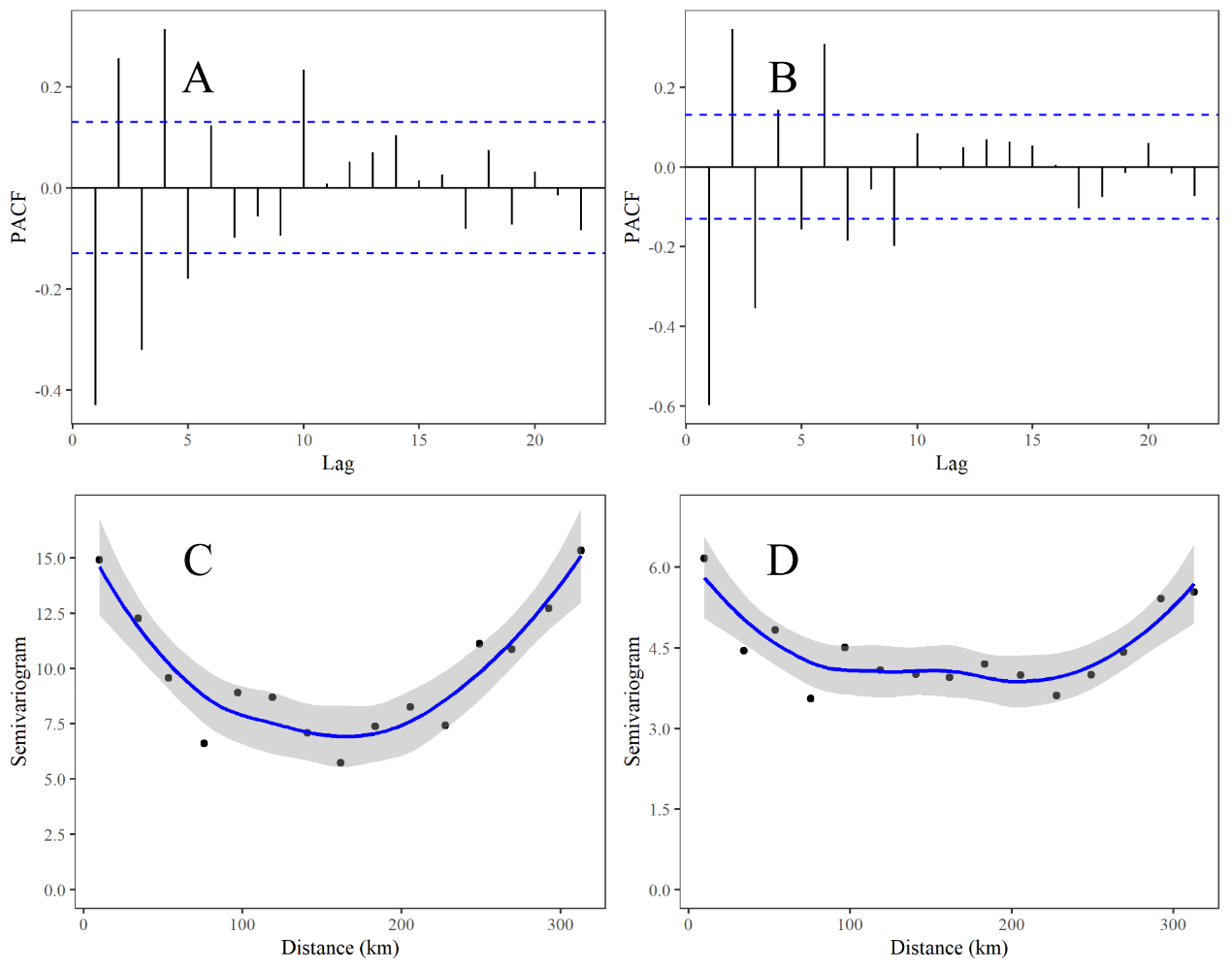
FIGURE S4 Partial autocorrelation function (PACF) of the yearly averages of the residual diagnostics and residual semivariogram diagnostics of the spatial correlation in the residuals for Brandt’s voles with and without interactive effects. A: PACF without interactive effects; B: PACF with interactive effects; C: Semivariogram without interactive effects; D: Semivariogram with interactive effects.


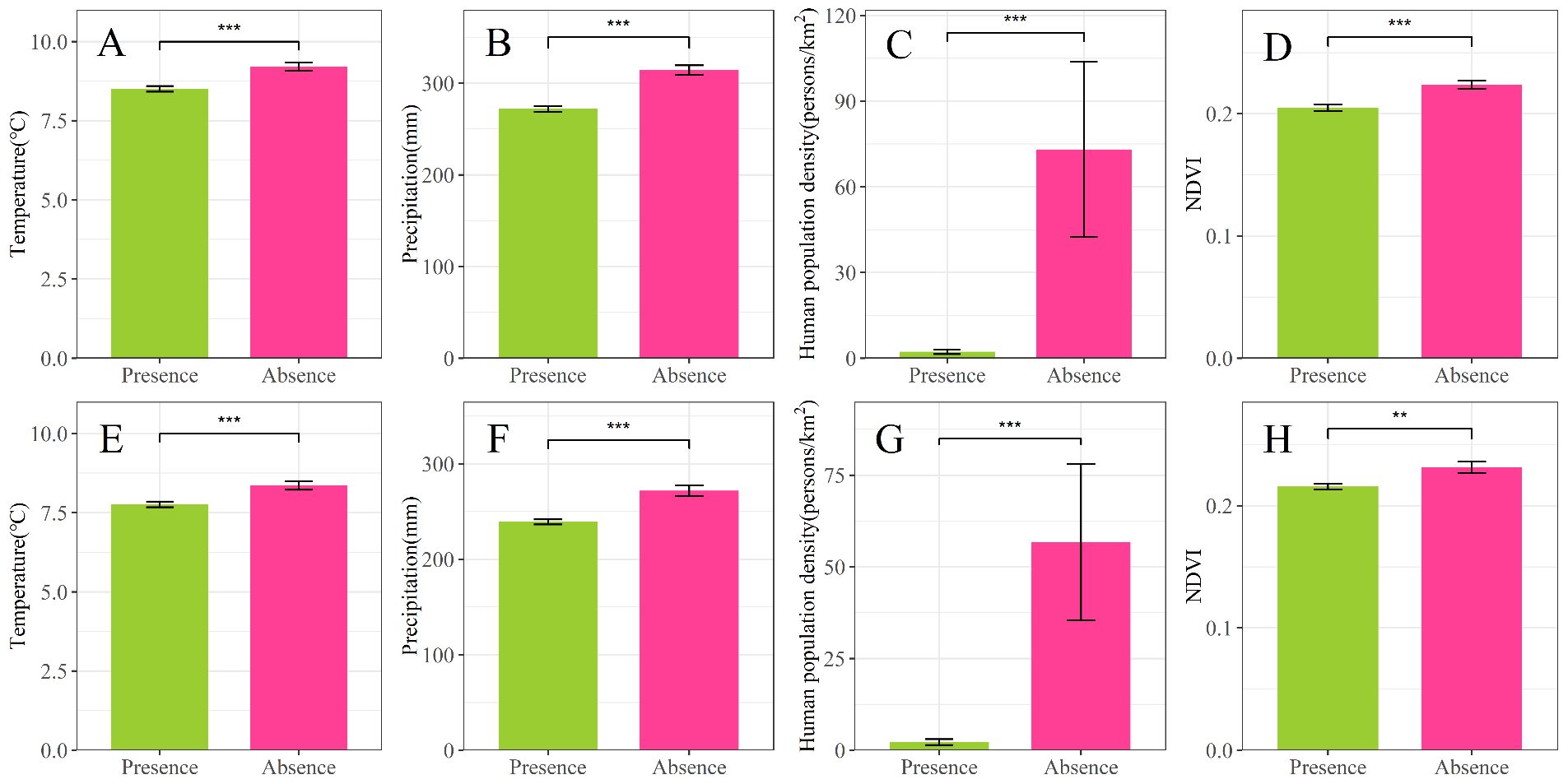
FIGURE S5 Difference of the most recent accessible environment variables between presence (0) and absence (1) grid-cells of Brandt’s voles (A: air temperature in 2018; B: precipitation in 2018; C: human population density in 2017; D: NDVI in 2015), and difference of environment variables of presence grid-cells during 2010–2018 and those of absence grid-cells 10-yrs before absence year (the year after last observation year) (E: air temperature; F: precipitation; G: human population density; H: NDVI). The green and red color of each histogram represented the average values of environment variables of different grid-cells. Error bars show the standard error (SE) (** *p* < 0.01; *** *p* < 0.001).

TABLE S1 Data resources used for getting presence data of Brandt’s voles in China, Mongolia and Russia. Due to the insufficient data in Mongolia and Russia, we only used presence records of Brandt’s voles in China for data filtering and statistical analysis.

| Data resources | Number of presence records in China | Number of presence records in Mongolia | Number of presence records in Russia |
| --- | --- | --- | --- |
| Distribution data of Brandt’s voles during 1970–2020 from online literatures using ISI Web of Knowledge (https://www.webofscience.com/), Google Scholar (https://www.scholar.google.com) and CNKI.net (https://www.cnki.net/) as search engines | 1984 | 161 | 12 |
| Global Biodiversity Information Facility (GBIF; https://www.gbif.org) | 63 | 162 | 6 |
| iNaturalist (https://www.inaturalist.org/) | 0 | 25 | 0 |
| Regular field monitoring and surveys of Brandt's voles in 45 locations on the Inner Mongolia grassland during 1993–2020 | 345 | 0 | 0 |
| Current distribution status reconfirmation investigation in September 2019 | 231 | 0 | 0 |

TABLE S2 Data verification of historical distribution data of Brandt’s voles from literatures and online databases in China. A total of 2042 presence records of Brandt’s voles were collected, and 534 records from historical literature and 40 records from Global Biodiversity Information Facility database were finally used (dark green color cells) after removing unqualified data (all grey-colored cells).

| Time and location resolution classifications | | Time resolution | | | | | |
| --- | --- | --- | --- | --- | --- | --- | --- |
|  |  | Historical literature | | | Global Biodiversity Information Facility (GBIF) database | | |
|  |  | Specific year records (e.g., "1998".) | Records not designated to specific year but with one decade (e.g., "1990S".) | Unclear records without time information | Specific year records (e.g., "1998".) | Records not designated to specific year but with one decade (e.g., "1990S".) | Unclear records without time information |
| Location resolution | Specific location with latitude and longitude recorded (e.g., Li et al. (2017) sampled Brandt's volesin Inner Mongolia with coordinates (42°25.61' N;115°16.33' E) reported.) | 232 | 0 | 0 | 40 | 0 | 0 |
|  | Specific location description which can be designated into specific grids of 10 × 10 km^2^ (e.g., village or ranch) | 304 | 0 | 0 | 0 | 0 | 0 |
|  | Presence information with only township description (e.g., "Narenbaolige Sumu") | 302 | 0 | 0 | 0 | 0 | 0 |
|  | Presence information with only county description (e.g., Zhangbei County) | 791 | 0 | 44 | 0 | 0 | 0 |
|  | Presence information with only league description (e.g., Xilingole League) | 176 | 0 | 10 | 0 | 0 | 0 |
|  | Presence information with only prefecture description (e.g., Inner Mongolia) | 74 | 0 | 20 | 11 | 0 | 2 |
|  | Presence information with only Country description (e.g., China) | 5 | 20 | 6 | 7 | 0 | 3 |
|  | Presence records without spatial information | 0 | 0 | 0 | 0 | 0 | 0 |

TABLE S3 Correlation analysis between independent variables.

|  | Standardized human population density | Standardized precipitation | Standardized temperature | Standardized NDVI |
| --- | --- | --- | --- | --- |
| Standardized human population density | 1 | 0.3483^***^ | 0.0882 | 0.1329 |
| Standardized precipitation | 0.3483^***^ | 1 | –0.2872^***^ | 0.3578^***^ |
| Standardized temperature | 0.0882 | –0.2872^***^ | 1 | –0.1489* |
| Standardized NDVI | 0.1329 | 0.3578^***^ | –0.1489^*^ | 1 |

^*^ *p* < 0.05, ^***^ *p* < 0.001

TABLE S4 Correlation of proportion of survived grids of Brandt’s voles with air temperature, human population density, precipitation and NDVI. The grid resolution is 10 × 10 km^2^.

|  | Correlation coefficients | | | |
| --- | --- | --- | --- | --- |
|  | Temperature | Human population density | Precipitation | NDVI |
| Proportion of survived grid-cells | –0.5127^***^ | –0.8886^***^ | 0.1492 | –0.0380 |

^***^ *p* < 0.001

TABLE S5 Number of grids with disappearance threshold of yearly maximum air temperature and maximum temperature of the warmest month for Brandt’s voles in the first absence year. The first absence year was defined as the first year after last observation time in each grid-cell. The disappearance threshold of yearly maximum air temperature was estimated to be 8.42±1.89 ℃. The disappearance threshold of maximum air temperature of the warmest month in the first absence year was 27.50±1.61 ℃.

| Yearly maximum air temperature range (℃) | Number of grid-cells | Maximum temperature of the warmest month (℃) | Number of grid-cells |
| --- | --- | --- | --- |
| 4.0–4.9 | 5 | 23.0–23.9 | 0 |
| 5.0–5.9 | 11 | 24.0–24.9 | 9 |
| 6.0–6.9 | 11 | 25.0–25.9 | 14 |
| 7.0–7.9 | 18 | 26.0–26.9 | 12 |
| 8.0–8.9 | 16 | 27.0–27.9 | 31 |
| 9.0–9.9 | 27 | 28.0–28.9 | 24 |
| 10.0–10.9 | 16 | 29.0–29.9 | 13 |
| 11.0–11.9 | 4 | 30.0–30.9 | 6 |
| 12.0–12.9 | 1 | 31.0–31.9 | 1 |
| 13.0–13.9 | 1 | – | – |

TABLE S6 Generalized additive models (GAM) selection with and without interactions between variables (popd_s: standardized HPD; prec_s: standardized precipitation; temp_s: standardized temperature; ndvi_s: standardized NDVI). Only significant interactive effects (i.e., interactions between standardized HPD and standardized precipitation, interactions between standardized HPD and NDVI, interactions between standardized precipitation and standardized temperature, interactions between standardized precipitation and NDVI) were included for model selection. s (Latitude, Longitude, k = 4) is the spatial autocorrelation effects. Models were ranked by smaller value of unbiased risk estimators (UBRE).

| Model | UBRE |
| --- | --- |
| Models without interactive effects |  |
| fate ~ popd_s + prec_s + temp_s + ndvi_s + s(Latitude_g, Longitude_g, k = 4) | 0.1137 |
| Models with significant interactive effects |  |
| fate ~ popd_s + prec_s + temp_s + ndvi_s + popd_s:prec_s + popd_s:ndvi_s + prec_s:temp_s + prec_s:ndvi_s + s(Latitude_g, Longitude_g, k = 4) | –0.0628 |
| fate ~ popd_s + prec_s + temp_s + ndvi_s + popd_s:prec_s + s(Latitude_g, Longitude_g, k = 4) | 0.0524 |
| fate ~ popd_s + prec_s + temp_s + ndvi_s + prec_s:temp_s + s(Latitude_g, Longitude_g, k = 4) | 0.0576 |
| fate ~ popd_s + prec_s + temp_s + ndvi_s + prec_s:ndvi_s + s(Latitude_g, Longitude_g, k = 4) | 0.0665 |
| fate ~ popd_s + prec_s + temp_s + ndvi_s + popd_s:ndvi_s + s(Latitude_g, Longitude_g, k = 4) | 0.0735 |

TABLE S7 Regular field survey on rodent abundance on the Inner Mongolia grassland during 1993–2020. The presence (0) or absence (1) status of Brandt’s vole was recorded for sites surveyed each year. Sites not investigated in a certain year were represented by “NA”.

| No | Longitude | Latitude | 1993 | 1994 | 1995 | 1996 | 1997 | 1998 | 1999 | 2000 | 2001 | 2002 | 2003 | 2004 | 2005 | 2006 | 2007 | 2008 | 2009 | 2010 | 2011 | 2012 | 2013 | 2014 | 2015 | 2016 | 2017 | 2018 | 2019 | 2020 |
| --- | --- | --- | --- | --- | --- | --- | --- | --- | --- | --- | --- | --- | --- | --- | --- | --- | --- | --- | --- | --- | --- | --- | --- | --- | --- | --- | --- | --- | --- | --- |
| 1 | 115.11 | 41.78 | 0 | 0 | 0 | 0 | 0 | 0 | 1 | 1 | 1 | 1 | 1 | 1 | 1 | 1 | 1 | 1 | 1 | 1 | 1 | 1 | 1 | 1 | 1 | 1 | 1 | 1 | 1 | 1 |
| 2 | 115.29 | 42.02 | 0 | 0 | 0 | 0 | 0 | 0 | 1 | 1 | 1 | 1 | 1 | 1 | 1 | 1 | 1 | 1 | 1 | 1 | 1 | 1 | 1 | 1 | 1 | 1 | 1 | 1 | 1 | 1 |
| 3 | 115.73 | 42.33 | 0 | 0 | 0 | 0 | 0 | 0 | 1 | 1 | 1 | 1 | 1 | 1 | 1 | 1 | 1 | 1 | 1 | 1 | 1 | 1 | 1 | 1 | 1 | 1 | 1 | 1 | 1 | 1 |
| 4 | 115.98 | 43.00 | NA | NA | 0 | 0 | 0 | 0 | 1 | 1 | 1 | 1 | 1 | 1 | 1 | 1 | 1 | 1 | 1 | 1 | 1 | 1 | 1 | 1 | 1 | 1 | 1 | 1 | 1 | 1 |
| 5 | 115.28 | 42.36 | NA | NA | 0 | 0 | 0 | 0 | 0 | 0 | 0 | 0 | 0 | 0 | 1 | 1 | 1 | 1 | 1 | 1 | 1 | 1 | 1 | 1 | 1 | 1 | 1 | 1 | 1 | 1 |
| 6 | 114.68 | 42.46 | NA | NA | 0 | 0 | 0 | 0 | 0 | 1 | 1 | 1 | 1 | 1 | 1 | 1 | 1 | 1 | 1 | 1 | 1 | 1 | 1 | 1 | 1 | 1 | 1 | 1 | 1 | 1 |
| 7 | 116.28 | 43.33 | NA | NA | NA | NA | NA | NA | NA | NA | NA | NA | NA | NA | NA | NA | NA | 0 | 0 | 0 | 0 | 1 | 1 | 1 | 1 | 1 | 1 | 1 | 1 | 1 |
| 8 | 116.67 | 43.63 | 0 | 0 | 0 | 1 | 1 | 1 | 1 | 1 | 1 | 1 | 1 | 1 | 1 | 1 | 1 | 1 | 1 | 1 | 1 | 1 | 1 | 1 | 1 | 1 | 1 | 1 | 1 | 1 |
| 9 | 116.57 | 43.97 | NA | NA | NA | NA | NA | 1 | 1 | 1 | 1 | 1 | 1 | 1 | 1 | 0 | 0 | 0 | 0 | 1 | 1 | 1 | 1 | 1 | 1 | 1 | 1 | 1 | 1 | 1 |
| 10 | 116.33 | 44.32 | NA | NA | NA | NA | NA | 1 | 1 | 1 | 1 | 1 | 1 | 1 | 1 | 0 | 0 | 0 | 0 | 1 | 1 | 1 | 1 | 1 | 1 | 1 | 1 | 1 | 1 | 1 |
| 11 | 115.74 | 44.81 | NA | NA | NA | NA | NA | 1 | 1 | 1 | 1 | 0 | 0 | 0 | 0 | 0 | 0 | 0 | 0 | 0 | 0 | 0 | NA | NA | NA | NA | NA | NA | NA | NA |
| 12 | 116.06 | 43.51 | NA | NA | NA | NA | NA | 1 | 1 | 1 | 1 | 1 | 1 | 1 | 1 | 1 | 1 | 0 | 0 | 0 | 0 | 0 | 1 | 1 | 1 | 1 | 1 | 1 | 1 | 1 |
| 13 | 114.70 | 44.89 | 0 | 0 | 0 | 0 | 0 | 0 | 0 | 0 | 0 | 0 | 0 | 0 | 0 | 0 | 0 | 0 | 0 | 0 | 0 | 0 | 0 | 0 | 0 | 0 | 0 | 0 | 0 | 0 |
| 14 | 114.11 | 44.62 | 0 | 0 | 0 | 0 | 0 | 0 | 0 | 0 | 0 | 0 | 0 | 0 | 0 | 0 | 0 | 0 | 0 | 0 | 0 | 0 | 0 | 0 | 0 | 0 | 0 | 0 | 0 | 0 |
| 15 | 115.35 | 44.79 | 0 | 0 | 0 | 0 | 0 | 0 | 0 | 0 | 0 | 0 | 0 | 0 | 0 | 0 | 0 | 0 | 0 | 0 | 0 | 0 | 0 | 0 | 0 | 0 | 0 | 0 | 0 | 0 |
| 16 | 115.33 | 45.10 | 0 | 0 | 0 | 0 | 0 | 0 | 0 | 0 | 0 | 0 | 0 | 0 | 0 | 0 | 0 | 0 | 0 | 0 | 0 | 0 | 0 | 0 | 0 | 0 | 0 | 0 | 0 | 0 |
| 17 | 112.57 | 44.74 | 0 | 0 | 0 | 0 | 0 | 0 | 0 | 0 | 0 | 0 | 0 | 0 | 0 | 0 | 0 | 0 | 0 | 0 | 0 | 0 | 0 | 0 | 0 | 0 | 0 | 0 | 0 | 0 |
| 18 | 116.38 | 45.02 | 0 | 0 | 0 | 0 | 0 | 0 | 0 | 0 | 0 | 0 | 0 | 0 | 0 | 0 | 0 | 0 | 0 | 0 | 0 | 0 | 0 | 0 | 0 | 0 | 0 | 0 | 0 | NA |
| 19 | 116.62 | 45.21 | NA | NA | NA | NA | NA | 0 | 0 | 0 | 0 | 0 | 0 | 0 | 0 | 0 | 0 | 0 | 0 | 0 | 0 | 0 | 0 | 0 | 0 | 0 | 0 | NA | NA | NA |
| 20 | 116.80 | 45.60 | NA | NA | NA | NA | NA | NA | NA | NA | NA | 0 | 0 | 0 | 0 | 0 | 0 | 0 | 0 | 0 | 0 | 0 | 0 | 0 | 0 | 0 | 0 | NA | NA | NA |
| 21 | 118.95 | 45.73 | NA | NA | NA | NA | NA | NA | NA | NA | NA | NA | NA | NA | NA | 0 | 0 | 0 | 0 | NA | NA | NA | NA | NA | NA | NA | NA | NA | NA | NA |
| 22 | 116.81 | 43.27 | 0 | 0 | 0 | 0 | 0 | 0 | 0 | 0 | 0 | 0 | 0 | 0 | 0 | 0 | 0 | 0 | 0 | 0 | 0 | 0 | 0 | 0 | 0 | NA | NA | NA | NA | NA |
| 23 | 116.90 | 43.34 | 0 | 0 | 0 | 0 | 0 | 0 | 1 | 1 | 1 | 1 | 1 | 1 | 1 | 1 | 1 | 1 | 1 | 1 | 1 | 1 | 1 | 1 | 1 | 1 | 1 | 1 | 1 | NA |
| 24 | 118.73 | 48.11 | NA | NA | NA | NA | NA | NA | NA | NA | NA | NA | NA | NA | NA | NA | NA | 0 | 0 | 0 | 0 | 0 | NA | NA | NA | NA | NA | NA | NA | NA |
| 25 | 118.98 | 48.64 | NA | NA | NA | NA | NA | NA | NA | NA | NA | NA | NA | NA | NA | NA | NA | 0 | 0 | 0 | 0 | 0 | NA | NA | NA | NA | NA | NA | NA | NA |
| 26 | 117.61 | 48.71 | NA | NA | NA | NA | NA | NA | NA | NA | NA | NA | NA | NA | NA | NA | NA | 0 | 0 | 1 | 1 | 1 | NA | NA | NA | NA | NA | NA | NA | NA |
| 27 | 117.33 | 48.31 | NA | NA | NA | NA | NA | NA | NA | NA | NA | NA | NA | NA | NA | NA | NA | 0 | 0 | 1 | 1 | 1 | NA | NA | NA | NA | NA | NA | NA | NA |
| 28 | 117.63 | 47.97 | NA | NA | NA | NA | NA | NA | NA | NA | NA | NA | NA | NA | NA | NA | NA | 0 | 0 | 0 | 0 | 0 | NA | NA | NA | NA | NA | NA | NA | NA |
| 29 | 116.87 | 48.62 | NA | NA | NA | NA | NA | NA | NA | NA | NA | NA | NA | NA | NA | NA | NA | 0 | 0 | 0 | 1 | 1 | NA | NA | NA | NA | NA | NA | NA | NA |
| 30 | 117.09 | 48.51 | NA | NA | NA | NA | NA | NA | NA | NA | NA | NA | NA | NA | NA | NA | NA | 0 | 0 | 0 | 0 | 0 | NA | NA | NA | NA | NA | NA | NA | NA |
| 31 | 115.98 | 48.18 | NA | NA | NA | NA | NA | NA | NA | NA | NA | NA | NA | NA | NA | NA | NA | 0 | 0 | 0 | 0 | 0 | NA | NA | NA | NA | NA | NA | NA | NA |
| 32 | 117.30 | 49.08 | NA | NA | NA | NA | NA | NA | NA | NA | NA | NA | NA | NA | NA | NA | NA | 0 | 0 | 0 | 1 | 1 | NA | NA | NA | NA | NA | NA | NA | NA |
| 33 | 117.27 | 49.39 | NA | NA | NA | NA | NA | NA | NA | NA | NA | NA | NA | NA | NA | NA | NA | 1 | 1 | 1 | 1 | 1 | NA | NA | NA | NA | NA | NA | NA | NA |
| 34 | 118.47 | 49.31 | NA | NA | NA | NA | NA | NA | NA | NA | NA |  | NA | NA | NA | NA | NA | 1 | 1 | 1 | 1 | 1 | NA | NA | NA | NA | NA | NA | NA | NA |
| 35 | 119.19 | 49.33 | NA | NA | NA | NA | NA | NA | NA | NA | NA | NA | NA | NA | NA | NA | NA | 0 | 0 | 0 | 0 | 0 | NA | NA | NA | NA | NA | NA | NA | NA |
| 36 | 119.92 | 48.94 | NA | NA | NA | NA | NA | NA | NA | NA | NA | NA | NA | NA | NA | NA | NA | 0 | 0 | 0 | 0 | 0 | NA | NA | NA | NA | NA | NA | NA | NA |
| 37 | 119.68 | 50.52 | 1 | 1 | 1 | 1 | 1 | 1 | 1 | 1 | 1 | 1 | 1 | 1 | 1 | 1 | 1 | 1 | 1 | 1 | 1 | 1 | 1 | 1 | 1 | 1 | 1 | 1 | 1 | 1 |
| 38 | 119.60 | 50.12 | 1 | 1 | 1 | 1 | 1 | 1 | 1 | 1 | 1 | 1 | 1 | 1 | 1 | 1 | 1 | 1 | 1 | 1 | 1 | 1 | 1 | 1 | 1 | 1 | 1 | 1 | 1 | 1 |
| 39 | 119.95 | 49.96 | 1 | 1 | 1 | 1 | 1 | 1 | 1 | 1 | 1 | 1 | 1 | 1 | 1 | 1 | 1 | 1 | 1 | 1 | 1 | 1 | 1 | 1 | 1 | 1 | 1 | 1 | 1 | 1 |
| 40 | 118.49 | 46.30 | 1 | 1 | 1 | 1 | 1 | 1 | 1 | 1 | 1 | 1 | 1 | 1 | 1 | 1 | 1 | 1 | 1 | 1 | 1 | 1 | 1 | 1 | 1 | 1 | 1 | 1 | 1 | 1 |
| 41 | 119.77 | 46.55 | 1 | 1 | 1 | 1 | 1 | 1 | 1 | 1 | 1 | 1 | 1 | 1 | 1 | 1 | 1 | 1 | 1 | 1 | 1 | 1 | 1 | 1 | 1 | 1 | 1 | 1 | 1 | 1 |
| 42 | 119.47 | 47.40 | 1 | 1 | 1 | 1 | 1 | 1 | 1 | 1 | 1 | 1 | 1 | 1 | 1 | 1 | 1 | 1 | 1 | 1 | 1 | 1 | 1 | 1 | 1 | 1 | 1 | 1 | 1 | 1 |
| 43 | 117.69 | 46.02 | 1 | 1 | 1 | 1 | 1 | 1 | 1 | 1 | 1 | 1 | 1 | 1 | 1 | 1 | 1 | 1 | 1 | 1 | 1 | 1 | 1 | 1 | 1 | 1 | 1 | 1 | 1 | 1 |
| 44 | 117.61 | 45.69 | 1 | 1 | 1 | 1 | 1 | 1 | 1 | 1 | 1 | 1 | 1 | 1 | 1 | 1 | 1 | 1 | 1 | 1 | 1 | 1 | 1 | 1 | 1 | 1 | 1 | 1 | 1 | 1 |
| 45 | 116.01 | 42.47 | 1 | 1 | 1 | 1 | 1 | 1 | 1 | 1 | 1 | 1 | 1 | 1 | 1 | 1 | 1 | 1 | 1 | 1 | 1 | 1 | 1 | 1 | 1 | 1 | 1 | 1 | 1 | 1 |

TABLE S8 Presence or absence data of Brandt’s voles during 1971–2020 used for GAM analysis. Data of two sampling years in each grid-cell was used. The first year after last observation (i.e., absence year of Brandt’s vole) and the year that was 10 years before the absence year were used as the two sampling years.

| Grid-cell code | Sampling year | Latitude of the grid-cell | Longitude of the grid-cell | Presence (0) or Absence (1) |
| --- | --- | --- | --- | --- |
| 0 | 1983 | 41.59903898 | 115.9705594 | 0 |
| 0 | 1993 | 41.59903898 | 115.9705594 | 1 |
| 1 | 1989 | 41.68236898 | 115.0539294 | 0 |
| 1 | 1999 | 41.68236898 | 115.0539294 | 1 |
| 2 | 1980 | 41.68236898 | 115.1372594 | 0 |
| 2 | 1990 | 41.68236898 | 115.1372594 | 1 |
| 3 | 1970 | 41.68236898 | 115.2205894 | 0 |
| 3 | 1980 | 41.68236898 | 115.2205894 | 1 |
| 4 | 1995 | 41.68236898 | 115.7205694 | 0 |
| 4 | 2005 | 41.68236898 | 115.7205694 | 1 |
| 5 | 1990 | 41.76569898 | 115.1372594 | 0 |
| 5 | 2000 | 41.76569898 | 115.1372594 | 1 |
| 6 | 1985 | 41.84902898 | 114.6372794 | 0 |
| 6 | 1995 | 41.84902898 | 114.6372794 | 1 |
| 7 | 1989 | 42.01568898 | 115.3039194 | 0 |
| 7 | 1999 | 42.01568898 | 115.3039194 | 1 |
| 8 | 1971 | 42.01568898 | 115.8872294 | 0 |
| 8 | 1981 | 42.01568898 | 115.8872294 | 1 |
| 9 | 1996 | 42.09901898 | 114.7206094 | 0 |
| 9 | 2006 | 42.09901898 | 114.7206094 | 1 |
| 10 | 1989 | 42.09901898 | 114.9705994 | 0 |
| 10 | 1999 | 42.09901898 | 114.9705994 | 1 |
| 11 | 1987 | 42.26567898 | 113.8873094 | 0 |
| 11 | 1997 | 42.26567898 | 113.8873094 | 1 |
| 12 | 1991 | 42.26567898 | 114.7206094 | 0 |
| 12 | 2001 | 42.26567898 | 114.7206094 | 1 |
| 13 | 1978 | 42.26567898 | 114.9705994 | 0 |
| 13 | 1988 | 42.26567898 | 114.9705994 | 1 |
| 14 | 1973 | 42.26567898 | 115.7205694 | 0 |
| 14 | 1983 | 42.26567898 | 115.7205694 | 1 |
| 15 | 1977 | 42.34900898 | 113.7206494 | 0 |
| 15 | 1987 | 42.34900898 | 113.7206494 | 1 |
| 16 | 1980 | 42.34900898 | 114.9705994 | 0 |
| 16 | 1990 | 42.34900898 | 114.9705994 | 1 |
| 17 | 1977 | 42.34900898 | 115.1372594 | 0 |
| 17 | 1987 | 42.34900898 | 115.1372594 | 1 |
| 18 | 1995 | 42.34900898 | 115.3039194 | 0 |
| 18 | 2005 | 42.34900898 | 115.3039194 | 1 |
| 19 | 1989 | 42.34900898 | 115.7205694 | 0 |
| 19 | 1999 | 42.34900898 | 115.7205694 | 1 |
| 20 | 1990 | 42.43233898 | 114.6372794 | 0 |
| 20 | 2000 | 42.43233898 | 114.6372794 | 1 |
| 21 | 2001 | 42.43233898 | 115.3039194 | 0 |
| 21 | 2011 | 42.43233898 | 115.3039194 | 1 |
| 22 | 1978 | 42.43233898 | 115.3872494 | 0 |
| 22 | 1988 | 42.43233898 | 115.3872494 | 1 |
| 23 | 1977 | 42.51566898 | 114.4706194 | 0 |
| 23 | 1987 | 42.51566898 | 114.4706194 | 1 |
| 24 | 1984 | 42.59899898 | 115.4705794 | 0 |
| 24 | 1994 | 42.59899898 | 115.4705794 | 1 |
| 25 | 1989 | 43.01564898 | 115.9705594 | 0 |
| 25 | 1999 | 43.01564898 | 115.9705594 | 1 |
| 26 | 2001 | 43.01564898 | 116.4705394 | 0 |
| 26 | 2011 | 43.01564898 | 116.4705394 | 1 |
| 27 | 1986 | 43.26563898 | 116.3872094 | 0 |
| 27 | 1996 | 43.26563898 | 116.3872094 | 1 |
| 28 | 2006 | 43.26563898 | 116.8038594 | 0 |
| 28 | 2016 | 43.26563898 | 116.8038594 | 1 |
| 29 | 1971 | 43.26563898 | 117.3038394 | 0 |
| 29 | 1981 | 43.26563898 | 117.3038394 | 1 |
| 30 | 2002 | 43.34896898 | 116.3038794 | 0 |
| 30 | 2012 | 43.34896898 | 116.3038794 | 1 |
| 31 | 1989 | 43.34896898 | 116.8871894 | 0 |
| 31 | 1999 | 43.34896898 | 116.8871894 | 1 |
| 32 | 1995 | 43.43229898 | 116.0538894 | 0 |
| 32 | 2005 | 43.43229898 | 116.0538894 | 1 |
| 33 | 1985 | 43.43229898 | 116.8038594 | 0 |
| 33 | 1995 | 43.43229898 | 116.8038594 | 1 |
| 34 | 2001 | 43.43229898 | 116.8871894 | 0 |
| 34 | 2011 | 43.43229898 | 116.8871894 | 1 |
| 35 | 1990 | 43.51562898 | 115.1372594 | 0 |
| 35 | 2000 | 43.51562898 | 115.1372594 | 1 |
| 36 | 2004 | 43.51562898 | 116.0538894 | 0 |
| 36 | 2014 | 43.51562898 | 116.0538894 | 1 |
| 37 | 2004 | 43.51562898 | 116.9705194 | 0 |
| 37 | 2014 | 43.51562898 | 116.9705194 | 1 |
| 38 | 1986 | 43.59895898 | 116.6371994 | 0 |
| 38 | 1996 | 43.59895898 | 116.6371994 | 1 |
| 39 | 2000 | 43.59895898 | 116.7205294 | 0 |
| 39 | 2010 | 43.59895898 | 116.7205294 | 1 |
| 40 | 1986 | 43.59895898 | 116.8871894 | 0 |
| 40 | 1996 | 43.59895898 | 116.8871894 | 1 |
| 41 | 1966 | 43.68228898 | 115.5539094 | 0 |
| 41 | 1976 | 43.68228898 | 115.5539094 | 1 |
| 42 | 1972 | 43.68228898 | 116.5538694 | 0 |
| 42 | 1982 | 43.68228898 | 116.5538694 | 1 |
| 43 | 1999 | 43.84894898 | 112.3873694 | 0 |
| 43 | 2009 | 43.84894898 | 112.3873694 | 1 |
| 44 | 2003 | 43.84894898 | 120.0537294 | 0 |
| 44 | 2013 | 43.84894898 | 120.0537294 | 1 |
| 45 | 1998 | 43.93227898 | 115.8038994 | 0 |
| 45 | 2008 | 43.93227898 | 115.8038994 | 1 |
| 46 | 1999 | 43.93227898 | 116.0538894 | 0 |
| 46 | 2009 | 43.93227898 | 116.0538894 | 1 |
| 47 | 2000 | 43.93227898 | 116.5538694 | 0 |
| 47 | 2010 | 43.93227898 | 116.5538694 | 1 |
| 48 | 1971 | 43.93227898 | 117.2205094 | 0 |
| 48 | 1981 | 43.93227898 | 117.2205094 | 1 |
| 49 | 1990 | 44.01560898 | 114.9705994 | 0 |
| 49 | 2000 | 44.01560898 | 114.9705994 | 1 |
| 50 | 2001 | 44.01560898 | 116.7205294 | 0 |
| 50 | 2011 | 44.01560898 | 116.7205294 | 1 |
| 51 | 1999 | 44.09893898 | 117.9704794 | 0 |
| 51 | 2009 | 44.09893898 | 117.9704794 | 1 |
| 55 | 2001 | 44.18226898 | 116.3872094 | 0 |
| 55 | 2011 | 44.18226898 | 116.3872094 | 1 |
| 56 | 2003 | 44.18226898 | 116.4705394 | 0 |
| 56 | 2013 | 44.18226898 | 116.4705394 | 1 |
| 57 | 2001 | 44.18226898 | 116.6371994 | 0 |
| 57 | 2011 | 44.18226898 | 116.6371994 | 1 |
| 61 | 2006 | 44.26559898 | 116.8038594 | 0 |
| 61 | 2016 | 44.26559898 | 116.8038594 | 1 |
| 65 | 2000 | 44.34892898 | 116.3038794 | 0 |
| 65 | 2010 | 44.34892898 | 116.3038794 | 1 |
| 66 | 1970 | 44.34892898 | 117.7204894 | 0 |
| 66 | 1980 | 44.34892898 | 117.7204894 | 1 |
| 67 | 1966 | 44.43225898 | 112.6373594 | 0 |
| 67 | 1976 | 44.43225898 | 112.6373594 | 1 |
| 68 | 1995 | 44.43225898 | 113.6373194 | 0 |
| 68 | 2005 | 44.43225898 | 113.6373194 | 1 |
| 71 | 1965 | 44.51558898 | 113.0540094 | 0 |
| 71 | 1975 | 44.51558898 | 113.0540094 | 1 |
| 76 | 1995 | 44.59891898 | 113.1373394 | 0 |
| 76 | 2005 | 44.59891898 | 113.1373394 | 1 |
| 77 | 1995 | 44.59891898 | 113.3873294 | 0 |
| 77 | 2005 | 44.59891898 | 113.3873294 | 1 |
| 84 | 1995 | 44.68224898 | 112.8040194 | 0 |
| 84 | 2005 | 44.68224898 | 112.8040194 | 1 |
| 102 | 1969 | 44.76557898 | 117.4704994 | 0 |
| 102 | 1979 | 44.76557898 | 117.4704994 | 1 |
| 103 | 1995 | 44.84890898 | 112.2207094 | 0 |
| 103 | 2005 | 44.84890898 | 112.2207094 | 1 |
| 113 | 2005 | 44.84890898 | 116.7205294 | 0 |
| 113 | 2015 | 44.84890898 | 116.7205294 | 1 |
| 123 | 1973 | 44.93223898 | 116.5538694 | 0 |
| 123 | 1983 | 44.93223898 | 116.5538694 | 1 |
| 124 | 1970 | 44.93223898 | 118.5537894 | 0 |
| 124 | 1980 | 44.93223898 | 118.5537894 | 1 |
| 173 | 2004 | 45.51554898 | 115.9705594 | 0 |
| 173 | 2014 | 45.51554898 | 115.9705594 | 1 |
| 174 | 2005 | 45.51554898 | 116.9705194 | 0 |
| 174 | 2015 | 45.51554898 | 116.9705194 | 1 |
| 175 | 2004 | 45.59887898 | 116.6371994 | 0 |
| 175 | 2014 | 45.59887898 | 116.6371994 | 1 |
| 177 | 1978 | 45.59887898 | 122.8869494 | 0 |
| 177 | 1988 | 45.59887898 | 122.8869494 | 1 |
| 178 | 2005 | 45.68220898 | 116.9705194 | 0 |
| 178 | 2015 | 45.68220898 | 116.9705194 | 1 |
| 179 | 1993 | 45.76553898 | 116.2205494 | 0 |
| 179 | 2003 | 45.76553898 | 116.2205494 | 1 |
| 180 | 2002 | 45.76553898 | 116.3038794 | 0 |
| 180 | 2012 | 45.76553898 | 116.3038794 | 1 |
| 181 | 2000 | 45.76553898 | 118.9704394 | 0 |
| 181 | 2010 | 45.76553898 | 118.9704394 | 1 |
| 182 | 1969 | 46.18218898 | 118.4704594 | 0 |
| 182 | 1979 | 46.18218898 | 118.4704594 | 1 |
| 200 | 2003 | 48.09877898 | 118.7204494 | 0 |
| 200 | 2013 | 48.09877898 | 118.7204494 | 1 |
| 201 | 2003 | 48.18210898 | 115.9705594 | 0 |
| 201 | 2013 | 48.18210898 | 115.9705594 | 1 |
| 210 | 1998 | 44.59891898 | 115.3872494 | 0 |
| 210 | 2008 | 44.59891898 | 115.3872494 | 1 |
| 212 | 2000 | 48.34876898 | 117.3038394 | 0 |
| 212 | 2010 | 48.34876898 | 117.3038394 | 1 |
| 216 | 1999 | 48.34876898 | 118.3871294 | 0 |
| 216 | 2009 | 48.34876898 | 118.3871294 | 1 |
| 217 | 1977 | 48.34876898 | 119.1370994 | 0 |
| 217 | 1987 | 48.34876898 | 119.1370994 | 1 |
| 220 | 2001 | 48.43209898 | 116.4705394 | 0 |
| 220 | 2011 | 48.43209898 | 116.4705394 | 1 |
| 222 | 1966 | 48.43209898 | 117.2205094 | 0 |
| 222 | 1976 | 48.43209898 | 117.2205094 | 1 |
| 223 | 1990 | 48.43209898 | 117.3038394 | 0 |
| 223 | 2000 | 48.43209898 | 117.3038394 | 1 |
| 231 | 1967 | 48.59875898 | 116.9705194 | 0 |
| 231 | 1977 | 48.59875898 | 116.9705194 | 1 |
| 233 | 2003 | 48.59875898 | 118.9704394 | 0 |
| 233 | 2013 | 48.59875898 | 118.9704394 | 1 |
| 239 | 1966 | 48.68208898 | 116.9705194 | 0 |
| 239 | 1976 | 48.68208898 | 116.9705194 | 1 |
| 240 | 2000 | 48.68208898 | 117.6371594 | 0 |
| 240 | 2010 | 48.68208898 | 117.6371594 | 1 |
| 246 | 1962 | 48.76541898 | 116.3872094 | 0 |
| 246 | 1972 | 48.76541898 | 116.3872094 | 1 |
| 252 | 1977 | 48.76541898 | 119.8870694 | 0 |
| 252 | 1987 | 48.76541898 | 119.8870694 | 1 |
| 259 | 2004 | 43.51562898 | 112.0540494 | 0 |
| 259 | 2014 | 43.51562898 | 112.0540494 | 1 |
| 263 | 1991 | 48.93207898 | 119.4704194 | 0 |
| 263 | 2001 | 48.93207898 | 119.4704194 | 1 |
| 264 | 1990 | 48.93207898 | 119.7204094 | 0 |
| 264 | 2000 | 48.93207898 | 119.7204094 | 1 |
| 265 | 2003 | 48.93207898 | 119.8870694 | 0 |
| 265 | 2013 | 48.93207898 | 119.8870694 | 1 |
| 269 | 1966 | 49.09873898 | 116.9705194 | 0 |
| 269 | 1976 | 49.09873898 | 116.9705194 | 1 |
| 270 | 2001 | 49.09873898 | 117.3038394 | 0 |
| 270 | 2011 | 49.09873898 | 117.3038394 | 1 |
| 272 | 1990 | 49.09873898 | 119.9703994 | 0 |
| 272 | 2000 | 49.09873898 | 119.9703994 | 1 |
| 274 | 1951 | 49.18206898 | 117.6371594 | 0 |
| 274 | 1961 | 49.18206898 | 117.6371594 | 1 |
| 275 | 1966 | 49.18206898 | 118.0538094 | 0 |
| 275 | 1976 | 49.18206898 | 118.0538094 | 1 |
| 276 | 1966 | 49.18206898 | 118.4704594 | 0 |
| 276 | 1976 | 49.18206898 | 118.4704594 | 1 |
| 277 | 1966 | 49.18206898 | 118.8871094 | 0 |
| 277 | 1976 | 49.18206898 | 118.8871094 | 1 |
| 278 | 1973 | 49.18206898 | 119.7204094 | 0 |
| 278 | 1983 | 49.18206898 | 119.7204094 | 1 |
| 281 | 2001 | 49.26539898 | 118.0538094 | 0 |
| 281 | 2011 | 49.26539898 | 118.0538094 | 1 |
| 282 | 1966 | 49.26539898 | 118.3037994 | 0 |
| 282 | 1976 | 49.26539898 | 118.3037994 | 1 |
| 288 | 1990 | 49.34872898 | 117.1371794 | 0 |
| 288 | 2000 | 49.34872898 | 117.1371794 | 1 |
| 289 | 1983 | 49.34872898 | 117.2205094 | 0 |
| 289 | 1993 | 49.34872898 | 117.2205094 | 1 |
| 305 | 1996 | 49.59871898 | 117.3871694 | 0 |
| 305 | 2006 | 49.59871898 | 117.3871694 | 1 |
| 311 | 1996 | 41.84902898 | 115.8038994 | 0 |
| 311 | 2006 | 41.84902898 | 115.8038994 | 1 |
